# Applying a New REFINE Approach in *Zymomonas mobilis* Identifies Novel sRNAs That Confer Improved Stress Tolerance Phenotypes

**DOI:** 10.3389/fmicb.2019.02987

**Published:** 2020-01-10

**Authors:** Katie Haning, Sean M. Engels, Paige Williams, Margaret Arnold, Lydia M. Contreras

**Affiliations:** ^1^McKetta Department of Chemical Engineering, The University of Texas at Austin, Austin, TX, United States; ^2^Department of Aerospace Engineering & Engineering Mechanics, The University of Texas at Austin, Austin, TX, United States; ^3^Department of Computer Science and Engineering, School of Engineering and Applied Sciences, University at Buffalo, Buffalo, NY, United States; ^4^Institute for Cellular and Molecular Biology, The University of Texas at Austin, Austin, TX, United States

**Keywords:** small RNA, regulatory RNA, strain engineering, bioinformatics, systems biology, RNA-seq, transcriptome

## Abstract

As global controllers of gene expression, small RNAs represent powerful tools for engineering complex phenotypes. However, a general challenge prevents the more widespread use of sRNA engineering strategies: mechanistic analysis of these regulators in bacteria lags far behind their high-throughput search and discovery. This makes it difficult to understand how to efficiently identify useful sRNAs to engineer a phenotype of interest. To help address this, we developed a forward systems approach to identify naturally occurring sRNAs relevant to a desired phenotype: RNA-seq Examiner for Phenotype-Informed Network Engineering (REFINE). This pipeline uses existing RNA-seq datasets under different growth conditions. It filters the total transcriptome to locate and rank regulatory-RNA-containing regions that can influence a metabolic phenotype of interest, without the need for previous mechanistic characterization. Application of this approach led to the uncovering of six novel sRNAs related to ethanol tolerance in non-model ethanol-producing bacterium *Zymomonas mobilis*. Furthermore, upon overexpressing multiple sRNA candidates predicted by REFINE, we demonstrate improved ethanol tolerance reflected by up to an approximately twofold increase in relative growth rate compared to controls not expressing these sRNAs in 7% ethanol (v/v) RMG-supplemented media. In this way, the REFINE approach informs strain-engineering strategies that we expect are applicable for general strain engineering.

## Introduction

With rising demands of efficiency and sustainability, the use of microbes as chemical factories is increasingly attractive. In bacteria, small RNAs (sRNAs) regulate cellular pathways, and metabolic engineers increasingly exploit them for engineering purposes (Chappell et al., 2013; Vazquez-Anderson and Contreras, 2013; Kang et al., 2014; Qi and Arkin, 2014; Haning et al., 2015; Leistra et al., 2019). sRNAs regulate mRNA and protein expression, typically by binding mRNA and blocking translation or changing stability (Storz et al., 2011). These sRNAs commonly have short imperfect base pairing with their targets and some require auxiliary elements to perform their regulatory role, such as Hfq in Gram-negative bacteria; other sRNAs such as *Escherichia coli* CsrB regulate cellular processes by binding or sequestering proteins (Storz et al., 2011). Many natural sRNAs have been found to respond to environmental signals and coordinate network responses in a variety of microorganisms with potential use in the production of biofuels including cyanobacteria (Georg et al., 2014; Song et al., 2014). Their short length (50–300 nt), dynamic nature, and multi-target impacts make them especially attractive for engineering complex phenotypes.

Much work has been done to engineer the industrially relevant ethanologenic organism *Zymomonas mobilis* to enhance production of lignocellulosic bioproducts (Wang et al., 2018). Traditional metabolic engineering methods have created strains capable of producing alternative products such as sorbitol, levan, glycerol, as well as lactic, gluconic, succinic, and acetic acids (Rogers et al., 2007) and strains able to metabolize sugars such as xylose and arabinose found abundantly in lignocellulosic hydrolysates (Zhang et al., 1995; Deanda et al., 1996). However, recent studies have begun to show the value of using sRNAs in the engineering of phenotypes of interest in *Z. mobilis* (Cho et al., 2014, 2017).

Current engineering efforts to use sRNAs focus primarily on the design of synthetic transcripts to knock down expression of specific mRNA targets, typically by blocking their ribosome binding sites (RBS) (Haning et al., 2015). These targeted knockdowns are useful for optimizing individual pathways but are limited in addressing complex phenotypes like stress tolerance, which involve large sets of genes (Wassarman, 2002).

While strategies for engineering natural sRNAs have been successful, they have been mostly limited to well-characterized pathways in model organisms. For example, the overexpression of naturally occurring sRNAs RprA, ArcZ, and DsrA has been shown to improve acid tolerance in *E. coli* (Gaida et al., 2013). Similarly, overexpression of sRNA RyhB in *E. coli* increased production of 5-aminolevulinic acid by 16% (Li et al., 2014). Other phenotypes improved by natural sRNA engineering strategies include succinate, fatty acid, amorphadiene, and butanol production (Kang et al., 2012; McKee et al., 2012; Jones et al., 2016). In these cases, the wealth of previous sRNA characterization (known mRNA targets and mechanisms) enabled engineers to foresee and achieve phenotype goals (Massé et al., 2007; Battesti et al., 2011). The contribution of regulatory RNAs in metabolic engineering has recently been reviewed (Leistra et al., 2019).

A number of existing tools and techniques locate sRNAs including QRNA, Intergenic Sequence Inspector, RNAz, sRNApredict/SIPHT, sRNA scanner, and nocoRNAc, and deep sequencing and identification of TSS (Pichon and Felden, 2003; Livny et al., 2005; Washietl et al., 2005; Sridhar et al., 2010; Herbig and Nieselt, 2011; Vockenhuber et al., 2011; Livny, 2012; Kaur and Balgir, 2018). But most rely on conservation of sequence and/or structure and depend on the set of known sRNAs and homology, which is often lacking in non-model organisms. Additionally, most of these programs are not readily available for current users. Recently, machine learning has been applied to identify bona fide sRNAs in multiple bacterial species based on intrinsic features in the genomic context of the sRNAs, which is more highly conserved across species compared to sRNA sequence (Eppenhof and Peña-Castillo, 2019). Still, the sRNA candidates predicted by these tools require experimental validation as they carry no evidence of actual transcript expression *in vivo*. From a list of candidates, researchers typically screen each by northern blot. Considering the low-throughput nature of Northern blotting analysis, many sRNA candidates from long lists may go untested. Upon identification of an sRNA with detectable expression, follow-up experiments may include knockout or overexpression to observe phenotype impacts and gel shift assays to check binding with any mRNA targets predicted by programs like IntaRNA or CopraRNA (Busch et al., 2008; Wright et al., 2013). Ultimately, the process is slow and lack direction toward metabolic engineering goals.

Expression-based approaches are more suitable to identify stress-responsive or phenotype-relevant sRNAs (Barquist and Vogel, 2015). For example, in the initial sRNA discovery efforts in *Z. mobilis* (Cho et al., 2014), visual inspection of transcriptome data yielded 95 sRNA candidates, and this led to the detection of expression of 15 sRNAs by Northern blotting. In this study, sequence-based approaches, WU-BLAST (Gish, 2002) and SIPHT (Livny, 2012), contributed 20 and 4 sRNA candidates, respectively. Only 10 of the 95 candidates identified by transcriptome data overlapped with the sequence search method sets. Ultimately, the sequence-based tools only contributed 2 of the 15 sRNAs verified by Northern blotting. This suggests the sequence-based tools, particularly in non-model organisms, leave many sRNAs undetected. Additionally, sRNAs that could contribute to a phenotype unique to an organism (like the high ethanol tolerance of *Z. mobilis*) may not prove widely conserved since the phenotype lacks conservation as well. sRNA candidates predicted by these tools require experimental validation. Although transcriptomics approaches do represent *in vivo* expression, noise from non-specific read mapping casts some doubt, and specific transcript ends prove difficult to discern without follow-up experiments.

Advances in these sequence search algorithms and in high-throughput sequencing have enabled discovery of hundreds of sRNAs across bacteria (Gelderman and Contreras, 2013; Tsai et al., 2015), but characterization lags far behind. The vast majority of sRNAs remain without any known function. Mechanistic characterization of these sRNAs requires low-throughput knockout and overexpression studies, a particular challenge in non-model organisms (Papenfort et al., 2008; Modi et al., 2011; Mars et al., 2015). For metabolic engineers, it has been impractical to consider the large (and growing) pool of sRNA regulators for specific goals since most sRNAs lack foreseeable roles in producing phenotypes of interest.

**FIGURE 1:**
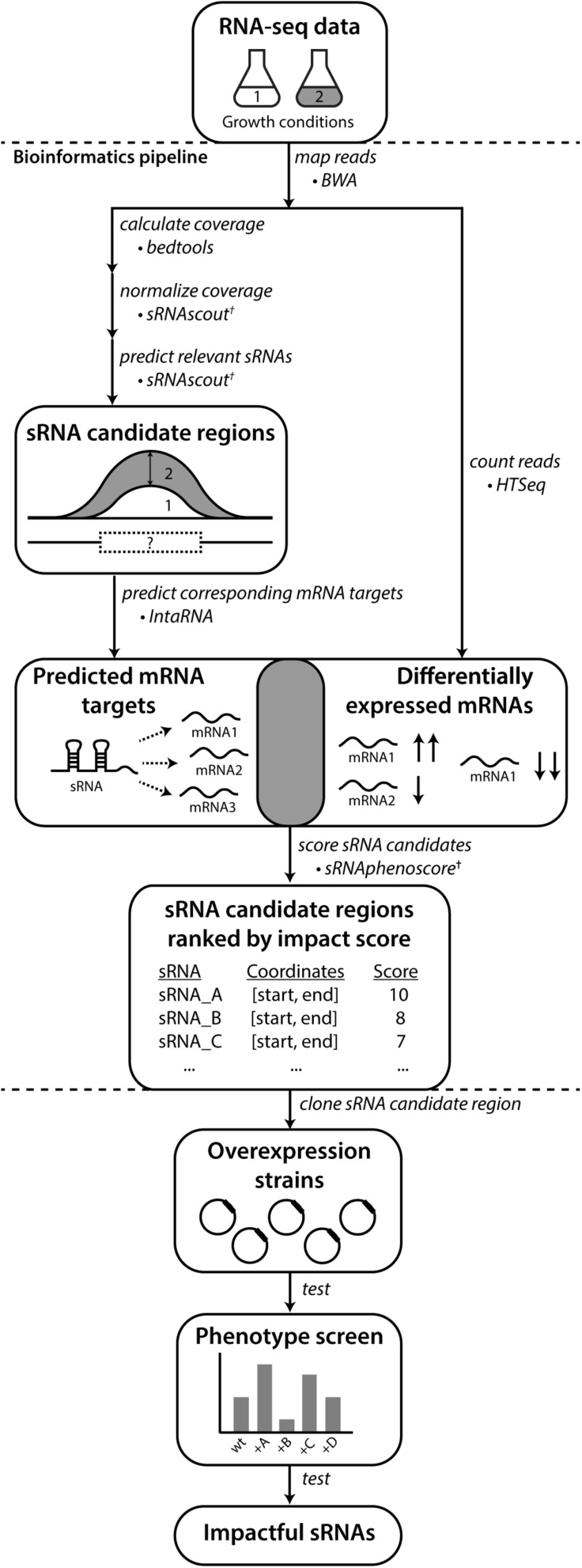
REFINE approach identifies sRNAs relevant to phenotypes of industrial interest. Existing computational tools were combined with new tools (†) developed in this work. Raw RNA-seq data from two conditions related to the phenotype of interest are analyzed for differentially expressed RNAs using standard tools for alignment (BWA) and counting (HTSeq). The REFINE sRNA prediction step identifies differentially expressed intergenic regions likely to contain sRNAs based on their length and a minimum expression level. For each sRNA candidate identified, IntaRNA predicts its most likely mRNA targets based on binding energies. Differential expression of these predicted mRNA targets between the two experimental conditions suggest possible regulation by the sRNA. A score is assigned to each sRNA based on the differential expression of its predicted targets and also the differential expression of the sRNA transcript itself. In this scoring equation, predicted mRNA targets with higher IntaRNA ranking contribute more heavily to the score. The calculation of a total score for each sRNA produces a ranked list of genomic regions most likely to contain sRNAs relevant to the phenotype. These sRNA candidates can each be overexpressed on plasmids and tested for their impact on the phenotype.

In this paper, we propose a bioinformatics pipeline, RNA-seq Examiner for Phenotype-Informed Network Engineering (REFINE) to identify sRNAs relevant to desired phenotypes using existing “omics” datasets (Figure 1). This systematic approach does not require previous sRNA characterization or discovery. Instead, we take advantage of RNA-seq datasets that accumulate in public databases and contain a wealth of insight into regulatory networks not yet extracted (Vogel and Marcotte, 2012). Importantly, we take advantages of potential connections between growth conditions and phenotypes documented in these studies that connect strain performance with RNA and protein expression profiles. In this particular work, we aim to exploit transcriptome data to predict regulatory networks and then characterize phenotypic impacts of predicted sRNA regulators. By prioritizing the list of sRNA candidates, we save experimental time and cost. To demonstrate the value of this new approach, we select industrially relevant phenotypes with known sRNAs involved in their regulation and RNA-seq data available. With this bioinformatics-informed approach, we show a more efficient way to find useful RNA regulators to construct a phenotype of interest, prioritizing the most promising sRNA candidates for any biochemical analysis.

## Materials and Methods

### Calculating Intergenic Region Coverage From Existing RNA-Seq Data

**FIGURE 2:**
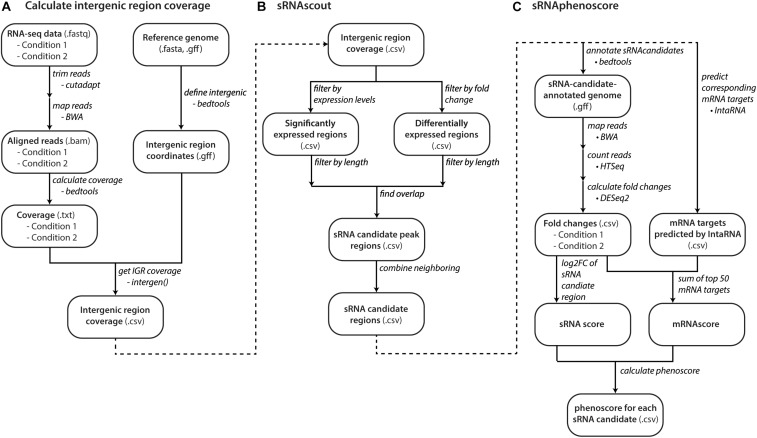
Computational workflow from raw RNA-seq data to phenoscores of each predicted sRNA candidate. **(A)** With raw RNA-seq data and a reference genome, files with coverage of intergenic regions are made for each growth condition. **(B)** sRNAscout filters intergenic regions down to short peak regions of minimum expression and differential expression between the two growth conditions. Neighboring peaks are combined into single regions that likely represent single transcripts. **(C)** The phenotypic impact of each sRNA candidate is predicted by sRNAphenoscore. The phenoscore is composed of two components: the sRNAscore and the mRNAscore. The sRNAscore is the absolute value of the log2 fold change of expression in the sRNA candidate region between the two growth conditions represented in the RNA-seq data. The mRNAscore is calculated by predicting the top 50 mRNA targets of each sRNA candidate by IntaRNA. It is calculated as the fold changes of each mRNA in the RNA-seq data between the two conditions scaled by its predicted energy from IntaRNA. The sRNAscore and mRNAscores are normalized by the max of each across all sRNA candidates and summed to produce the final phenoscore for each sRNA candidate.

RNA-seq data from previous studies were analyzed as shown in Figure 2A. Raw fastq files were downloaded from NCBI SRA (*Z. mobilis* aerobic and anaerobic: SRR1291412-3). Note, the compared conditions should be taken from a single experiment and RNAseq analysis. Read quality was visualized by FastQC (Andrews, 2016) and cutadapt (v1.3) trimmed any low-quality read ends (Martin, 2011). Burrows–Wheeler Aligner – maximal exact matches (BWA-mem) (v0.7.12-r1039) mapped the reads to the appropriate reference genome (*Z. mobilis* ZM4 ASM710v1) (Li, 2013). SAMtools sorted the aligned reads by name (v0.1.19-44428cd) (Li et al., 2009) and BEDTools (v2.25.0) calculated per-nucleotide genome coverages for each strand (±) of each sample (Quinlan and Hall, 2010). BEDTools also generated intergenic region coordinate lists from each reference genome. An R script produced comma-separated files describing the per-nucleotide read counts in the intergenic regions on each strand. Biological replicates (when available) were averaged to make a single file for each growth condition.

### sRNA Prediction With sRNAscout

sRNAscout was developed to predict sRNA-containing regions from these intergenic region coverage files (Figure 2B). First, it normalizes coverage files by read count. Then it filters by minimum expression level (in at least one condition) and differential expression level (between the two conditions). sRNAscout extracts consecutive nucleotides of sufficient length meeting these criteria as sRNA candidate peaks. Any peaks <200 nt are expanded outward and combined with any neighboring peaks to yield sRNA candidate regions. Appropriate values for these criteria vary by dataset according to overall depth of coverage, read quality, and rRNA depletion. We suggest 15 nt length for minimum expression and differential expression. For the levels of expression and differential expression, we suggest the averages of each across the dataset. Table 1 shows the values used in this study.

**TABLE 1:** Filtering criteria used by sRNAscout to identify sRNA candidate peak regions in each dataset.

**Criteria**	***Z. mobilis* aerobic vs. anaerobic**
Minimum expression level	34 counts
Consecutive nt with minimum expression	24 nt
Differential expression level	2.7-fold
Consecutive nt with differential expression	15 nt

### Scoring sRNA Candidates for Phenotype Effect With sRNAphenoscore

sRNAphenoscore (Figure 2C) identifies which sRNA candidate regions most likely impact the phenotype of interest. BEDTools added the predicted regions from sRNAscout to the reference genome and HTSeq (v0.6.1p1) counted the reads mapped (BWA) to each sRNA candidate region (Anders et al., 2015). DESeq2 calculates an sRNAscore for each candidate region from the absolute value of the log2 fold change between the conditions (Love et al., 2014). For each sRNA candidate region, IntaRNA (v2.2.0) predicts mRNA targets with most favorable binding energies (kcal/mol) in the −200 to +100 nt regions (Busch et al., 2008). To save computational time, we precomputed the accessibility data for each genome. sRNAphenoscore calculated an mRNAscore for each sRNA candidate region: the sum of the top 50 mRNA target energies multiplied by their log2 fold changes by DESeq2. The sRNAscores and mRNAscores for all sRNA candidate regions were normalized by the max of each, yielding a value between 0 and 1. sRNAphenoscore sums the sRNAscore and mRNAscore for each sRNA candidate to produce the final phenoscore for each.

### sRNA Candidate Overexpression Strain Development

To generate sRNA overexpression strains, GenScript synthesized and cloned each sRNA into the pBBR1MCS2-P_gap_ plasmid (Zou et al., 2012) between *Nhe*I and *Bam*HI. Each were transformed into *E. coli* DH5α and *Z. mobilis* 8b by electroporation. For the multi-sRNA overexpression strains, each sRNA was preceded by a P_gap_ promoter region in the pBB1MCS2 plasmid. *Z. mobilis* 8b grew in rich media supplemented with glucose (RMG) media (glucose, 20.0 g/L; yeast extract, 10.0 g/L; KH_2_PO_4_, 2.0 g/L; pH 6.0) at 33°C. *E. coli* DH5α (used for plasmid construction and manipulation) grew in LB media at 37°C. Strains containing the plasmids were cultured with 50 μg/mL kanamycin for *E. coli* and with 350 μg/mL for *Z. mobilis*. Transformants were screened by colony PCR and sequence-verified by Sanger sequencing.

### Evaluating Strain Performance

Biological triplicates of each strain were grown in 5 mL RMG seed cultures with appropriate antibiotics at 33°C for 48 h. Cells were distributed in technical triplicates into Bioscreen C (Growth Curves US, NJ, United States) plates with RMG (with and without 8% ethanol or 12 g/L sodium acetate) such that each well had a total volume of 300 μL and initial OD_600_ of 0.05. The Bioscreen C measured the turbidity with the wideband filter (420–580 nm) every 15 min for 48 h. The cultures grew at 33°C with 5 s of low-speed shaking before each measurement. EZ Experiment (Norden Logic Oy, Helsinki, Finland) operated the Bioscreen C and a custom MATLAB script calculated exponential growth rates (Crook et al., 2016). Additionally, in order to analyze growth conditions in larger culture volumes, seed cultures were used to inoculate 30 mL of RMG (with and without 7% ethanol) such that each culture had a starting OD_600_ of 0.05. The cultures were grown at 33°C with no shaking, and turbidity was measured every 3 h by adding 200 μL of culture into a 96-well plate and taking an OD_600_ measurement in a Cytation 3 (BioTek Winooski, VT, United States). Growth rates were then determined from the slope of the semilogarithmic plot of the OD_600_ readings versus time. Although *Z. mobilis* has been reported to tolerate up to 16% ethanol, our experiments have been performed at lower starting concentrations of ethanol to allow us to acquire consistent growth data without inhibition. The different assays performed in our experiments at 7 and 8% were performed in different conditions, growth in a flask with a 30-mL culture volume versus growth in a Bioscreen C plate with a 300-μL volume, respectively. We could not acquire consistent data using 8% ethanol in the flasks as we could in the Bioscreen C.

## Results

### Development of REFINE Approach to Identify sRNAs That Affect Phenotypes of Interest

We developed a bioinformatics pipeline to identify sRNA candidates most likely to impact a phenotype of interest. In this case study, we focus on ethanol tolerance (for *Z. mobilis*) as a novel test case. As shown in Figure 1, the input to the bioinformatics process is RNA-seq data from two growth conditions, based on the phenotype goal. For example, if the engineering goal were to develop a strain tolerant to a high-temperature industrial process, RNA would be collected and sequenced at a high temperature (stressed condition) and at its optimal growth temperature (unstressed condition). The comparison of these conditions allows observation of how the transcriptome changes naturally as part of the cellular response to the changing condition (or stress). As an underlying design principle of this analysis, we expect that with maximal stress the transcriptome response exhibited is more robust (as long as the imposed stress condition doesn’t hinder survival) and therefore the variability in RNA levels becomes significant enough to draw conclusions.

Public databases such as the Sequence Read Archive (SRA) of NCBI contain an abundance of RNA-seq data (Leinonen et al., 2011). RNA-seq data used with the REFINE pipeline should not be depleted of small transcripts during RNA extraction and library preparation (although rRNA depletion is appropriate). To determine which direction sRNAs are encoded in the genome, a strand-specific RNA-seq library prep is required (Wang et al., 2009). Considering the noise that can occur in RNA-seq datasets, biological replicates increase the reliability of the output. After experimental data collection, BWA maps the transcriptome data to a reference genome, yielding a landscape of transcript read abundance across the genome (Figure 2A). Then, the calculation of per-nucleotide coverage represents the number of reads mapped to each genomic location. We normalize coverage by each sample’s total read count to allow averaging of replicates and comparison between conditions.

The sRNAscout program (Figure 2B), a new algorithm developed in this work, uses the normalized per-nucleotide coverage files from each condition to predict genomic regions most likely to contain sRNAs that affect a particular phenotype of interest – we call these sRNA candidate regions. The sRNAscout program replaces the visual inspection and manual scrolling through transcriptome as used in the past. The primary advantage of this approach over other sequence-homology sRNA prediction tools is the targeted nature of the search for sRNAs that respond *in vivo* to changing environmental conditions (as represented by the two sets of input transcriptome data). Additionally, sRNAscout can identify more unique sRNAs that lack homology to those found in model organisms.

sRNAscout first filters the total transcriptome down to only transcripts mapping to intergenic regions, where RNA- and protein-encoding genes have not been annotated and where regulatory sRNAs have been found abundant. This step depends on a reference genome file including known mRNA, rRNA, tRNA, and any other transcripts that should not be classified as “intergenic” for the sake of sRNA prediction. The reference genome used here must contain the direction of each known transcript along with its genomic coordinates.

Next, sRNAscout searches intergenic regions for sRNA candidate peaks, defined by a sufficient, minimum expression level and differential expression. Previous sRNA identification work defines these criteria as important hallmarks of true sRNAs (Cho et al., 2014; Tsai et al., 2015). sRNAscout combines peak regions within 50 nt of each other into a single sRNA candidate region, since these likely constitute a single transcript. The program expands any short peak regions to 200 nt with the peak as the center.

From these potential sRNA-containing regions, we aim to discern which most likely regulate genes associated with the target phenotype. We hypothesize an sRNA linked to the phenotype will exhibit two main features. First, we expect the sRNA expression level to change between the two representative growth conditions, in the presence and absence of the stress of interest. The sRNAscore of the algorithm quantifies this feature as the magnitude of the log2 fold change in expression level between these conditions. Second, we expect the sRNA’s potential mRNA targets to show differential expression between the two conditions (in the presence and absence of the stress). The mRNAscore quantifies this.

Specifically, to determine mRNA targets that are likely regulated by the sRNA candidates, we employ an RNA–RNA interaction prediction program (IntaRNA) to yield a list of mRNAs in the organism with the most favorable binding energies to the sRNA (Wright et al., 2014). Among the available tools for sRNA target prediction, IntaRNA shows the highest accuracy without requiring the input of homologous sRNAs in other organisms (Pain et al., 2015). Benchmarking of IntaRNA showed the median rank of experimentally verified targets was 10 (Pain et al., 2015). Here, we use the top 50 mRNA targets predicted by IntaRNA for each sRNA candidate to quantify the potential impact of the sRNA on the expression of these mRNA predicted targets. We scale the magnitude of the log2 fold expression change of each predicted mRNA target between the two growth conditions (with and without the stress) by its predicted free energy of binding with the sRNA candidate. The sum of these scaled values for the top 50 mRNA targets represents the mRNAscore.

Finally, the program combines the sRNAscore and mRNAscore into a total phenoscore for each sRNA candidate (Figure 2C), in which a higher score indicates a region more likely to contain an sRNA that affects the phenotype of interest. As a result, the bioinformatics pipeline produces a list of sRNA candidate regions ranked by their phenoscore.

To experimentally verify the most promising candidates, we generated a library of overexpression strains, each containing an sRNA candidate region, and screened these strains for phenotype impact. We concluded that sRNA overexpression strains with significantly different performance compared to the wild type (or to an empty plasmid control) reveal useful sRNA candidates that can contribute toward engineering of a phenotype of interest. In this way, we expect to inform strain engineering strategies in which we overexpress sRNAs that enhance a particular phenotype and knock down sRNAs that negatively impact the phenotype.

### Identification of sRNAs in *Z. mobilis* That Improve Ethanol Tolerance

We aimed to engineer ethanol tolerance in *Zymomonas mobilis* by identifying novel sRNAs. *Zymomonas mobilis* tolerates up to 16% (v/v), making it an especially interesting bacterium for biofuel applications (Rogers et al., 2007; Yang et al., 2016a; Wang et al., 2018). Over the last 20 years, metabolic engineering and directed evolution developed a variety of *Z. mobilis* strains (Zhang et al., 1995; Mohagheghi et al., 2014). Transcriptome data are available for *Z. mobilis* under a variety of stresses including ethanol, furfural, acetate, and oxygen (Yang et al., 2009; He et al., 2012; Yang et al., 2013, 2014; Cho et al., 2014). Although ethanol stress RNA-seq data is not available for *Z. mobilis* (only microarray transcriptomes), anaerobic and aerobic data have been collected. Anaerobic conditions facilitate higher levels of ethanol production relative to aerobic conditions (Bringer et al., 1984; Moreau et al., 1997) and therefore are routinely used to represent conditions of lower and higher ethanol, respectively.

Recently, the discovery of 15 sRNAs in *Z. mobilis* included four responsive in expression to oxygen or ethanol stresses, representing potential regulators for engineering robustness (Cho et al., 2014). However, the low-throughput process of identifying these sRNAs included manual searches through transcriptome data, followed by northern blotting. This study demonstrates a systematic approach to identify sRNAs specifically useful to enhance ethanol tolerance.

**FIGURE 3:**
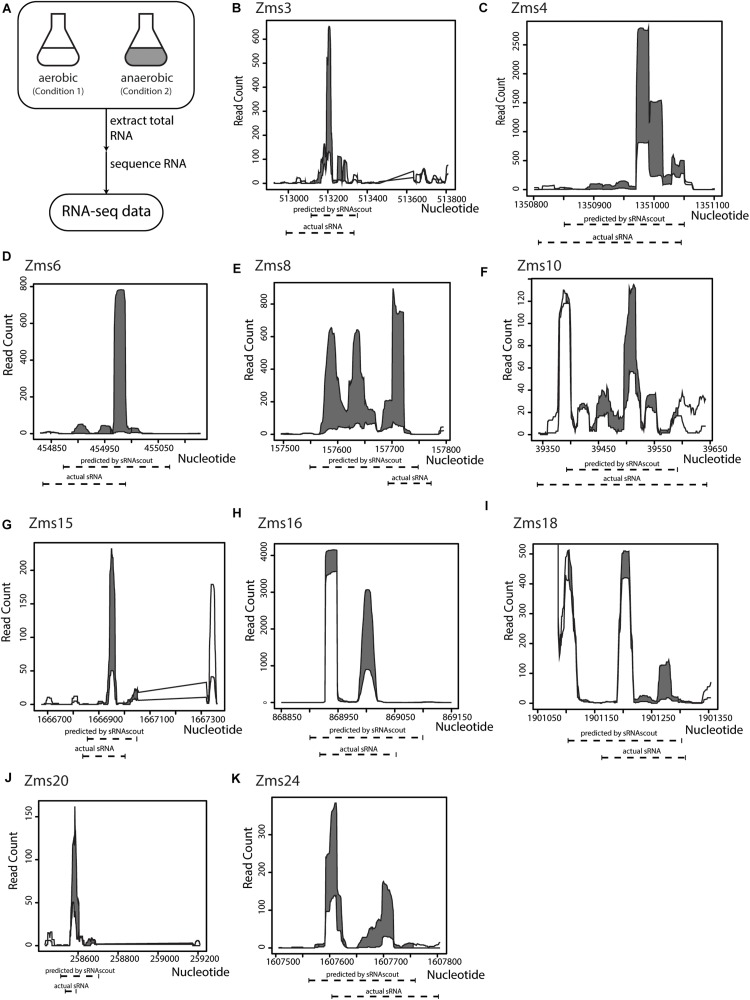
sRNAscout identifies previously known *Z. mobilis* sRNAs responsive to oxygen stress. **(A)** In a previous study, *Z. mobilis* ZM4 was grown in aerobic and anaerobic conditions and the RNA was extracted and sequenced (Cho et al., 2014). Original manual analysis of these data identified 13 sRNAs which were verified by northern blot. In this study, these data were analyzed with the REFINE approach to find the sRNAs automatically. **(B–K)** The expression profile of each known sRNA is shown for the aerobic and anaerobic conditions, as well as the predicted coordinates by sRNAscout compared to experimentally verified ends.

To identify ethanol-enhancing sRNAs in *Z. mobilis* in a high-throughput manner, we processed the raw RNA-seq files and used sRNAscout to predict sRNAs (Figure 3A). With the filtering criteria (Table 1), sRNAscout predicted 679 sRNA candidate regions (Supplementary Table S1). Rfam was used to predict homology of these candidates to known RNAs, and any hits were listed in Supplementary Table S1. We found that there was little homology, and the majority of hits reside in the tRNA family. However, homology is not always informative for identifying sRNAs in non-model organisms such as *Z. mobilis*, meaning there could be numerous real sRNAs in this predicted list. This *Z. mobilis* data retain rRNA and therefore exhibit more shallow sequencing depth for non-rRNA transcripts which led us to use less stringent expression criteria. Of the previously confirmed sRNAs in *Z. mobilis*, sRNAscout identified all as sRNA candidates except Zms2 [as a note, Zms13 and Zms14 (ENA ASM710v1) have now been annotated as tmRNA and CRISPR, respectively]. Figure 3 shows the expression profiles of these sRNAs and the specific regions predicted by sRNAscout. Note, sRNAscout probably failed to detect Zms2 because it shows the lowest expression and least differential expression among these verified sRNAs. As shown in Figure 3, the sRNA candidate genomic coordinates predicted by sRNAscout generally correlate with sRNA transcript ends experimentally verified by RACE (Cho et al., 2014).

Using IntaRNA, we next predicted the targets for each sRNA candidate region and the top 50 predictions (lowest energy) were used to calculate the mRNAscore. As shown in Table 2, the phenoscores of previously verified sRNAs showed a range comparable to the overall range of all sRNA candidates predicted by sRNAscout, suggesting a variety of potential impacts on the ethanol tolerance phenotype. It is worth noting that the contribution of the sRNAscore (SD = 0.153) seems more indicative of the overall phenoscore for this subset of verified sRNAs compared to the mRNAscore, which shows less variability (SD = 0.045).

**TABLE 2:** Phenoscores from aerobic vs. anaerobic data of previously discovered *Z. mobilis* sRNAs.

**sRNA**	**mRNAscore**	**sRNAscore**	**Phenoscore**	**Rank**	**5′-end**	**3′-end**
Zms6	0.58	0.781	1.361	16	454669	454972
Zms8	0.67	0.535	1.205	53	157766	157687
Zms20	0.785	0.406	1.191	70	258449	258569
Zms18	0.751	0.351	1.101	133	1901203	1900964
Zms3	0.705	0.167	0.873	347	512975	513362
Zms24	0.639	0.226	0.864	360	1607606	1607830
Zms4	0.719	0.101	0.82	427	1351044	1350765
Zms16	0.661	0.142	0.802	465	868928	869052
Zms10	0.639	0.125	0.764	520	39274	39493
Zms15	0.688	0	0.688	617	1666899	1666996

*These ranks correspond to the phenoscores across all 679 sRNA candidates predicted by sRNAscout in these conditions.*

**FIGURE 4:**
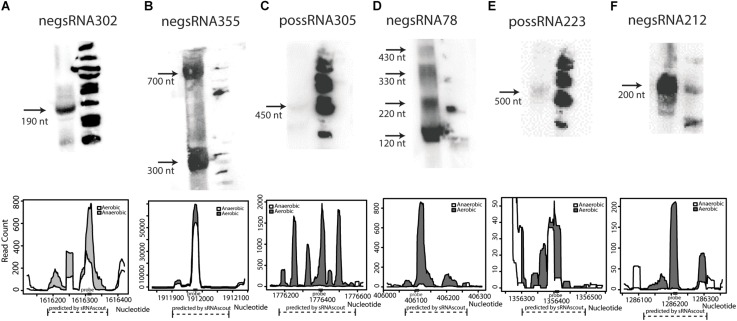
Novel sRNAs in *Z. mobilis* discovered by sRNAscout and confirmed by northern blot **(A–F)**. *Z. mobilis* sRNA candidates among the top 50 by phenoscore in aerobic vs. anaerobic conditions were tested by northern blot to verify detectable expression. In each blot, the first lane includes 30 μg total RNA of wild-type *Z. mobilis* 8b grown in anaerobic conditions and collected in stationary phase. The second lane includes ΦX174 DNA/*Hin*fI dephosphorylated markers. Each confirmed sRNA is shown along with its expression profile according to the RNA-seq data. A dashed line marks the genomic region predicted as an sRNA candidate by sRNAscout and a solid rectangle marks the location of the complementary probe designed for northern blotting.

Of the 50 sRNA candidates with highest phenoscores, 8 were tested by northern blot in a previous study and only Zms6 was detected (Cho et al., 2014). Thirty additional sRNAs were selected and tested for expression by northern blot. Six were confirmed to have detectable expression in stationary phase (Figure 4 and Supplementary Data Sheet S1), representing novel transcripts that are differentially expressed in response to ethanol stress.

**FIGURE 5:**
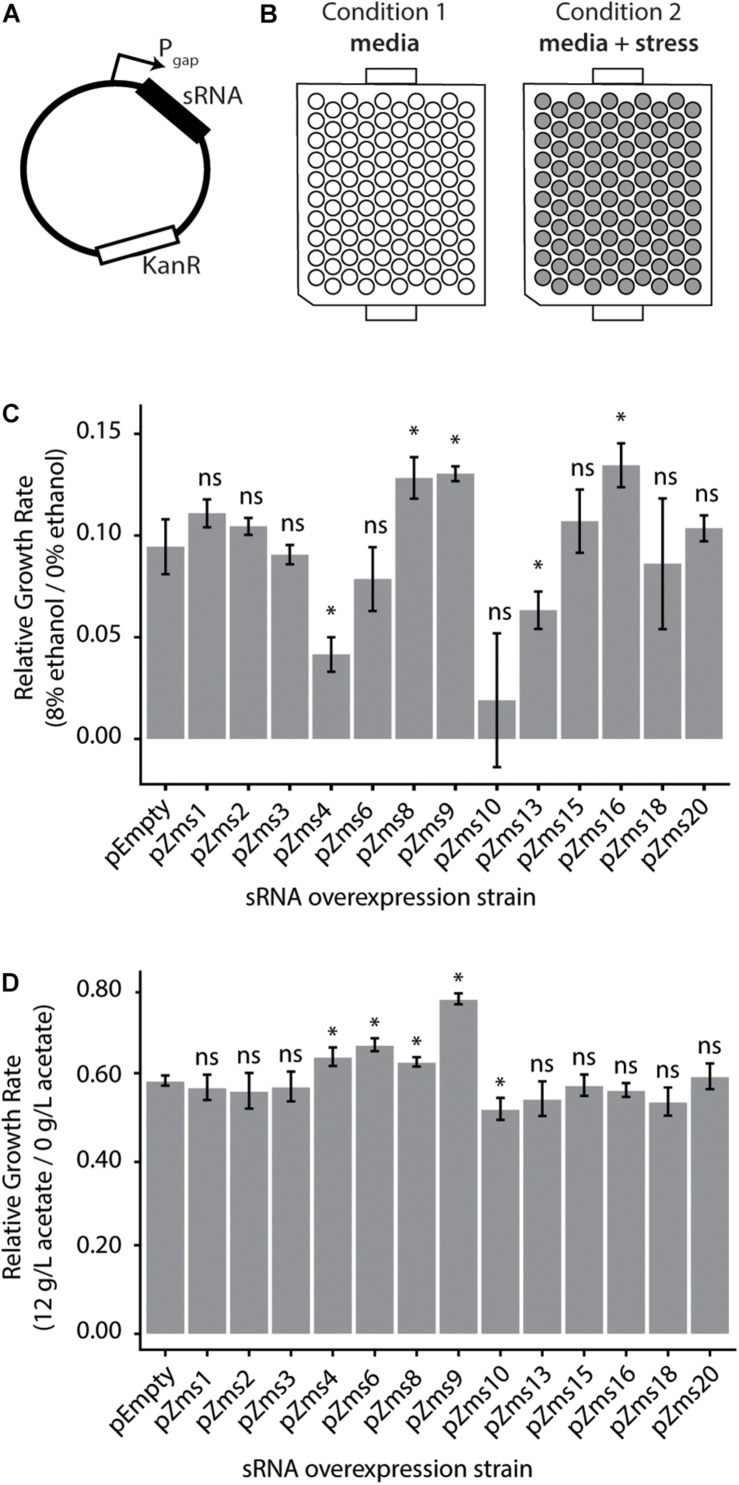
*Z. mobilis* sRNAs identified by the REFINE process impact stress tolerance. **(A)** Each previously identified *Z. mobilis* sRNA was cloned into a plasmid under the constitutive P_gap_ promoter for overexpression. **(B)** *Z. mobilis* strains each carrying an sRNA overexpression plasmid were grown in 300 μL plate cultures in two conditions: media only and media with added stress conditions (8% v/v ethanol or 12 g/L acetate). Cell density (OD_600_) was measured every 15 min for 24 h by the BioscreenC and maximum growth rates were calculated. **(C,D)** Growth rates of each strain were normalized to their own growth rate in the no-stress condition (gray bars, error bars = SD, *n* = 3). The relative growth rate of each overexpression strain was compared by *t*-test to the pEmpty plasmid strain which lacks an sRNA (^∗^*p* < 0.05). Multiple sRNAs show significant impact in 8% v/v ethanol stress **(C)** and in 12 g/L sodium acetate stress **(D)**.

To test the impact of these sRNAs on the ethanol-tolerance phenotype, we next cloned sRNAs (sequences given in Supplementary Table S2) into plasmids for overexpression under the constitutive Pgap promoter (Zou et al., 2012) (native to *Z. mobilis*, Figure 5A). Additionally, to observe the impacts of sRNAs with a range of phenoscores, we cloned the previously discovered sRNAs (ranging from 16 to 617 in the ranked phenoscore list, Supplementary Table S1). The strains were screened for their growth rates with and without 8% (v/v) ethanol supplemented to the media (Figure 5B). As shown in Figure 5C, the overexpression of Zms4 and Zms13 negatively impact growth rate in ethanol compared with the pEmpty control, harboring the same Pgap promoter plasmid in the absence of any sRNA overexpression. In contrast, overexpression strains of Zms8, Zms9, and Zms16 (Supplementary Table S2) show higher relative growth rates relative to the same pEmpty strain control. Among the set of sRNAs screened here, Zms8 has both the highest phenoscore as well as a significant positive impact on growth in ethanol stress.

To discern if these sRNAs respond specifically to ethanol or could be involved in other stress responses, these overexpression strains were grown in acetate stress as well (Figure 5D). Along with the desired sugar monomers, acetic acid is released during the industrial pretreatment process of cellulose and is a strong growth inhibitor (Mills et al., 2009). Only overexpression of Zms4, Zms6, Zms8, and Zms9 increased growth rate up to ∼30% while overexpression of Zms10 significantly decreased growth rate in acetate compared to the control strain. These results suggest that Zms4, Zms8, and Zms9 (but not Zms16) may play general stress response roles and be beneficial for a number of strain engineering goals.

Lastly, following the analysis of the previously discovered and experimentally confirmed sRNAs that were predicted by this analysis to be relevant to the engineering of an enhanced ethanol tolerance phenotype, the six newly discovered sRNAs confirmed by Northern blotting analysis (Figure 4) were also screened for their impact on growth rate. Importantly, we identified that overexpression of one additional newly identified sRNA (negsRNA302, Supplementary Table S2) increased growth rate by 43% in 7% (v/v) ethanol supplemented RMG relative to the control strain harboring the pEmpty plasmid.

**FIGURE 6:**
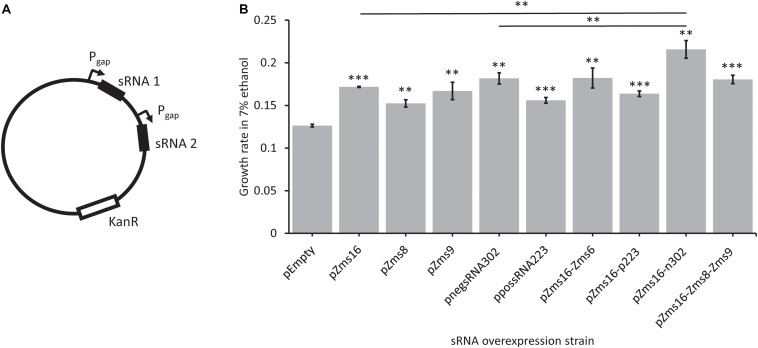
Novel sRNAs identified through REFINE impact *Z. mobilis* ethanol stress tolerance. Newly identified sRNAs in *Z. mobilis* were cloned into plasmids under the P_gap_ promoter for overexpression. Multi-overexpression plasmids were created with multiple sRNAs being expressed under separate P_gap_ promoters **(A)**. Strains were grown under stress in RMG supplemented with 7% ethanol. The relative growth rate of each overexpression strain was compared by *t*-test to the pEmpty plasmid strain which lacks an sRNA (error bars = SD, *n* = 3, ^∗^*p* < 0.05, ^∗∗^*p* < 0.01, ^∗∗∗^*p* < 0.001) **(B)**.

Multi-sRNA overexpression plasmids were also constructed (Figure 6A) to determine if a combinatorial effect could be seen by overexpressing multiple sRNAs that positively impacted growth alone. Zms16 was co-overexpressed with both negsRNA302 and possRNA223, and Zms16, Zms8, and Zms9 were also overexpressed together. Zms16, 8, and 9 were chosen for co-overexpression based upon their improvement in ethanol tolerance (Figure 5). We also chose to include overexpression of Zms6 with Zms16 as it was the known sRNA with the highest predicted phenoscore (Table 2). The highest performing of these sRNAs, Zms16, was paired with the two newly discovered sRNAs that improved tolerance, negsRNA302 and possRNA223. Importantly, we identified that co-overexpression of negsRNA302 and Zms16 further increased growth rate by approximately twofold in 7% (v/v) ethanol supplemented RMG when compared to the pEmpty control. Interestingly, the growth rate increase exhibited by the strain overexpressing the negsRNA302/Zms16 combination is higher than strains overexpressing negsRNA302 and Zms16 alone (Figure 6B). This result indicates that there could be a super additive effect on ethanol tolerance by over-expressing both sRNAs. It is worth noting that only this specific combination positively impacts growth rates under 7% (v/v) ethanol supplemented RMG as other combinations of Zms16 overexpression with other sRNAs did not results in a similar enhancement. The overexpression of Zms16 with Zms6, which showed no significant change in growth under ethanol stress (Figure 5), together showed similar increase in growth when Zms16 is expressed alone (Figure 6B).

## Discussion

The REFINE approach exploits transcriptome data and computational sRNA prediction tools to inform the engineering of complex phenotypes that exploit the tuning of intracellular sRNA levels. A user guide for this pipeline has been included in Supplementary Section 1.3. The bioinformatics process includes a novel pipeline that we have established, sRNAscout, for identifying sRNAs that impact the phenotype of interest without the need for previous functional characterization. By applying this pipeline in this study, we identified six novel sRNAs in *Z. mobilis* and we confirm their cellular expression via Northern blotting analysis. Additionally, we introduce a new pipeline, sRNAphenoscore, that assigns each sRNA candidate predicted by sRNAscout a score representing its predicted impact on the target phenotype. In this way, we demonstrate prioritizing the list of predicted sRNAs for follow-up characterization to those most likely useful for engineering strains with improved ethanol tolerance.

It is worth noting that when comparing results obtained for *Z. mobilis* from the sRNAscout algorithm to results obtained by the existing SIPHT algorithm (Livny, 2012), which has been used by several groups for the prediction of novel sRNA candidates (Khoo et al., 2012; Cho et al., 2014; Tsai et al., 2015), SIPHT predicted only four candidate regions, and only one of the two experimentally validated SIPHT regions (Genomic coordinates: 1079429-1080003, Zms1) was also found by sRNAscout. This comparison illustrates that sequence-based and transcriptome-based approaches predict different kinds of sRNA candidates. We also suspect that the sRNAscout tool offers a narrower pool of potential sRNAs of interest, targeting the list of identified transcripts to those that could most likely impact a phenotype of interest.

As strain engineering increasingly moves to fully automated systems, an approach like REFINE is attractive. With transcriptome data from two or more growth conditions, REFINE can computationally identify impactful genomic regions that can be targeted for genetic manipulations. Mutant strains can be cloned and screened in large libraries to find the most impactful mutations and then explore which combinations produce the fittest strain. Note that for high-throughput-automated strain development, an expression-based approach like sRNAscout, which finds any/all impactful genomic regions for mutation and screening, is preferable to a purely sequence-based approach. This is because it can identify any intergenic transcripts expressed differentially under the stress conditions, including undiscovered transcripts that may not be sRNAs, whereas a sequence-based approach would exclude these other non-sRNA discoveries.

A challenge we foresee for full automation is the identification of transcript ends for cloning. As shown in Figure 3, sRNAscout ends do not fully align with ends discerned by RACE in previous studies. sRNAscout identifies peaks of expression and, if <200 nt, the program expands the sRNA candidate region to 200 nt. For the transcripts to be functional as regulators, they must include the binding sites of their targets and exhibit sufficiently similar folding with the native transcripts. To identify the ends of sRNA candidates, the user may employ a computational approach like DeepBound (Shao et al., 2017) or a traditional experimental approach like 5′- and 3′-RACE. In some cases, it may be feasible to clone and test the entire intergenic region or to split it into multiple short fragments for testing. This will depend on the volume of cloning and screening that can be done and on the depth of sRNA characterization desired (and already existing for that organism). Of the two most impactful sRNAs predicted by REFINE, that have also been previously characterized, Zms16 and Zms8, the transcript lengths predicted by sRNAscout were longer and did not contain one end of Zms8. If the ends of negsRNA302 were to be more stringently characterized, overexpression could yield a larger positive impact on growth under ethanol stress assuming the native sRNA sequence was not fully captured by sRNAscout. Additionally, we predict that if the impactful sRNAs were to be encoded for overexpression in the genome, without use of plasmids, the fitness of the strains could be further advanced.

It is important to note that the sRNAs discovered by REFINE may or may not be direct regulators of the phenotype of interest. The native regulatory landscape is very complex; as such, some sRNAs may regulate multiple stresses while others play a very specific role. This bioinformatics approach is designed to predict the direct impacts because it scores sRNA candidates based on the direct binding of sRNA–mRNA pairs predicted by IntaRNA. But the differential expression of these mRNA targets could arise from direct sRNA regulation, indirect regulation, or no real regulation at all. It is impossible to decouple these things without more data; however, what this analysis validates if that this level of mechanistic detail is ultimately not necessary to screen and identify sRNAs that can be manipulated to affect a specific phenotype of interest. We hypothesize the two newly discovered sRNAs identified in this study that impacted ethanol tolerance, negsRNA302 and possRNA223, play regulatory roles in pathways relevant to ethanol tolerance, and could have implications in the genetic engineering for improvement of ethanologenic strains in the future. In the case of the *Z. mobilis* sRNA overexpression strains constructed and screened here using previously discovered sRNAs, we found that overexpression of Zms16 showed impact specific to ethanol stress whereas overexpression of Zms8 and Zms9 had a positive impact on growth rate under both ethanol and acetate stress. Interestingly, overexpression of Zms4 from this promoter only significantly positively impacted growth rates under acetate stress and indeed negatively impacted growth rate under ethanol stress. Additionally, sRNAs may work together with other sRNAs or regulators to coordinate the complex phenotype, this was seen through the beneficial increase in growth rates under ethanol stress observed from the co-overexpression of both Zms16 and negsRNA302.

Depending on the organism and available genomic manipulation methods, sRNA candidate overexpression on a plasmid may be altered using a new promoter or replaced by genomic overexpression, deletion, or knockdown. It is important to note that overexpression on a plasmid removes the transcript from its native regulatory context and may not give the full picture of an sRNA candidate’s phenotypic impact. The overexpression of some of these sRNAs under the inducible P_tet_ promoter in the pEZ plasmid (Yang et al., 2016b) has also been studied. An unpublished work in progress (Han et al., under review) found that overexpressing combinations of Zms4/Zms6, Zms4/Zms16, and Zms4/Zms6/Zms16 in this plasmid can positively impact growth of *Z. mobilis* in 6% ethanol, indicating that Zms4 could be co-overexpressed with other sRNAs to have a positive impact on growth. However, Zms16/Zms6 overexpression did not show a positive effect as it has in this study using the pBB1MCS2 plasmid. However, the focus of this work is a new method of high-throughput strain engineering, and thus investigating the impact of multiple plasmids and promoters on the desired phenotype does not contribute to the overarching goal. Considering the desired application of strain engineering (rather than sRNA characterization), transcripts that can be successfully manipulated to achieve a new phenotype carry the most value, so those very sensitive to additional layers of regulation for their activity may not be as desirable.

Overall, we expect that the REFINE method presented in this work can be widely applicable for strain engineering as increasing availability of RNA-seq data and accessibility of high-performance computing resources continue to increase for organisms of biotechnological interest.

## Data Availability Statement

The raw datasets generated in this study can be found in the Gene Expression Omnibus, accession numbers GSM1388375 and GSM1388376. The raw fastq files were downloaded from NCBI SRA [*Z. mobilis* aerobic and anaerobic: SRR1291412-3 (https://trace.ncbi.nlm.nih.gov/Traces/sra/?run=SRR1291412)].

## Author Contributions

KH and SE wrote the manuscript and performed the wet lab experiments. KH, PW, and MA designed the REFINE bioinformatic pipeline. LC provided the direction and guidance for the project.

## Conflict of Interest

The authors declare that the research was conducted in the absence of any commercial or financial relationships that could be construed as a potential conflict of interest.

## Supplementary Material

TABLE S1 sRNA candidate regions for *Z. mobilis* oxygen stress sRNA candidate regions predicted by sRNAscout and scored by phenoscoreCalc.

TABLE S2 sRNA sequences used for testing in ethanol stress assays.

DATA SHEET S1 Full images of northern blots for each confirmed sRNA identified by REFINE. In each blot, the first lane includes 30 μg total RNA of wild-type *Z. mobilis* 8b grown in anaerobic conditions and collected in stationary phase. The second lane includes ΦX174 DNA/HinfI Dephosphorylated Markers. **(A)** negsRNA302, **(B)** negsRNA355, **(C)** possRNA305, **(D)** negsRNA78, **(E)** possRNA223, and **(F)** negsRNA212.
