# Supplementary material for: Applying a New REFINE Approach in Zymomonas mobilis Identifies Novel sRNAs That Confer Improved Stress Tolerance Phenotypes
Source: Front Microbiol. 2020 Jan 10;10:2987. doi: 10.3389/fmicb.2019.02987 (PMC6970203; doi:10.3389/fmicb.2019.02987)
Supplement: TABLE S1 — sRNA candidate regions for Z. mobilis oxygen stress sRNA candidate regions predicted by sRNAscout and scored by phenoscoreCalc. [file Data_Sheet_2.docx]

Supplementary Material

# Supplementary Methods

## Supplementary Table 1.

| **Supplementary Table S1. sRNA candidate regions for** *Z. mobilis* oxygen stress | | | | | | |  |
| --- | --- | --- | --- | --- | --- | --- | --- |
| sRNA candidate regions predicted by sRNAscout and scored by phenoscoreCalc | | | | | |  |  |
|  |  |  |  |  |  |  |  |
| **sRNA** | **sRNAscore** | **mRNAscore** | **phenoscore** | **start** | **end** | **strand** | **known RNAs (Rfam)** |
| negsRNA119 | 1.000 | 0.595 | 1.595 | 556193 | 556393 | - |  |
| possRNA122 | 0.749 | 0.760 | 1.508 | 744976 | 745176 | + |  |
| possRNA7 | 0.749 | 0.757 | 1.507 | 49895 | 50095 | + |  |
| negsRNA286 | 0.561 | 0.928 | 1.490 | 1546468 | 1546668 | - |  |
| possRNA274 | 0.794 | 0.682 | 1.476 | 1633731 | 1633931 | + |  |
| negsRNA233 | 0.710 | 0.764 | 1.474 | 1159638 | 1159838 | - |  |
| negsRNA302 | 0.675 | 0.790 | 1.465 | 1616170 | 1616370 | - | tRNA |
| negsRNA261 | 0.490 | 0.943 | 1.433 | 1423902 | 1424102 | - |  |
| possRNA308 | 0.629 | 0.781 | 1.409 | 1791895 | 1792095 | + |  |
| possRNA305 | 0.574 | 0.835 | 1.409 | 1776171 | 1776580 | + |  |
| possRNA76 | 0.609 | 0.800 | 1.409 | 518114 | 518314 | + |  |
| possRNA77 | 0.609 | 0.790 | 1.399 | 527636 | 527836 | + |  |
| negsRNA301 | 0.550 | 0.847 | 1.397 | 1616017 | 1616217 | - |  |
| negsRNA355 | 0.532 | 0.845 | 1.377 | 1911885 | 1912090 | - | 5S_rRNA |
| negsRNA333 | 0.697 | 0.677 | 1.375 | 1796580 | 1796780 | - |  |
| Zms6 | 0.580 | 0.781 | 1.361 | 454961 | 45500 | - |  |
| possRNA100 | 0.524 | 0.827 | 1.352 | 622360 | 622560 | + |  |
| possRNA103 | 0.525 | 0.821 | 1.346 | 622736 | 622936 | + |  |
| possRNA304 | 0.712 | 0.629 | 1.341 | 1775982 | 1776182 | + |  |
| possRNA231 | 0.425 | 0.910 | 1.334 | 1374154 | 1374354 | + |  |
| negsRNA70 | 0.402 | 0.930 | 1.332 | 372219 | 372419 | - |  |
| negsRNA122 | 0.499 | 0.824 | 1.323 | 573674 | 573874 | - |  |
| negsRNA176 | 0.616 | 0.700 | 1.317 | 749946 | 750146 | - |  |
| possRNA223 | 0.596 | 0.715 | 1.311 | 1356300 | 1356500 | + |  |
| possRNA229 | 0.419 | 0.885 | 1.304 | 1373490 | 1373690 | + |  |
| negsRNA175 | 0.661 | 0.641 | 1.302 | 749848 | 750048 | - |  |
| negsRNA38 | 0.457 | 0.838 | 1.295 | 232530 | 232730 | - |  |
| negsRNA44 | 0.534 | 0.755 | 1.289 | 234876 | 235076 | - |  |
| negsRNA310 | 0.551 | 0.736 | 1.287 | 1644315 | 1644515 | - |  |
| possRNA212 | 0.455 | 0.830 | 1.286 | 1286106 | 1286306 | + |  |
| negsRNA78 | 0.458 | 0.823 | 1.281 | 406062 | 406262 | - |  |
| possRNA273 | 0.503 | 0.776 | 1.279 | 1623560 | 1623760 | + |  |
| negsRNA202 | 0.550 | 0.729 | 1.278 | 971258 | 971458 | - |  |
| negsRNA63 | 0.497 | 0.781 | 1.278 | 317431 | 317631 | - | tRNA |
| possRNA21 | 0.488 | 0.784 | 1.273 | 151281 | 151481 | + |  |
| negsRNA217 | 0.334 | 0.933 | 1.266 | 1073447 | 1073647 | - |  |
| negsRNA75 | 0.493 | 0.772 | 1.265 | 405412 | 405637 | - |  |
| negsRNA256 | 0.369 | 0.893 | 1.261 | 1386312 | 1386512 | - |  |
| negsRNA180 | 0.494 | 0.762 | 1.256 | 849424 | 849624 | - |  |
| negsRNA74 | 0.634 | 0.614 | 1.248 | 405159 | 405359 | - |  |
| negsRNA43 | 0.401 | 0.846 | 1.247 | 234184 | 234384 | - |  |
| negsRNA251 | 0.406 | 0.841 | 1.247 | 1368170 | 1368370 | - |  |
| possRNA13 | 0.406 | 0.841 | 1.247 | 118321 | 118521 | + |  |
| negsRNA212 | 0.581 | 0.661 | 1.242 | 1028554 | 1028754 | - | tRNA |
| negsRNA324 | 0.435 | 0.806 | 1.241 | 1746207 | 1746407 | - |  |
| possRNA2 | 0.485 | 0.756 | 1.240 | 12581 | 12781 | + |  |
| negsRNA37 | 0.571 | 0.668 | 1.239 | 232359 | 232559 | - |  |
| possRNA14 | 0.537 | 0.692 | 1.229 | 135565 | 135765 | + |  |
| possRNA114 | 0.389 | 0.833 | 1.222 | 691696 | 691896 | + | tRNA |
| possRNA238 | 0.390 | 0.829 | 1.219 | 1387667 | 1387867 | + |  |
| negsRNA338 | 0.537 | 0.669 | 1.206 | 1826992 | 1827192 | - |  |
| negsRNA209 | 0.368 | 0.837 | 1.205 | 1007849 | 1008049 | - |  |
| Zms8 | 0.535 | 0.670 | 1.205 | 157564 | 157717 | - |  |
| negsRNA45 | 0.506 | 0.699 | 1.205 | 235161 | 235387 | - |  |
| possRNA260 | 0.494 | 0.710 | 1.205 | 1563960 | 1564160 | + |  |
| possRNA157 | 0.514 | 0.688 | 1.202 | 966480 | 966680 | + |  |
| possRNA12 | 0.438 | 0.762 | 1.200 | 117379 | 117579 | + |  |
| negsRNA76 | 0.461 | 0.737 | 1.198 | 405765 | 405965 | - |  |
| possRNA245 | 0.354 | 0.842 | 1.197 | 1461364 | 1461564 | + |  |
| negsRNA232 | 0.490 | 0.705 | 1.195 | 1159477 | 1159677 | - |  |
| possRNA101 | 0.311 | 0.884 | 1.195 | 622480 | 622680 | + |  |
| possRNA232 | 0.392 | 0.803 | 1.195 | 1374321 | 1374521 | + |  |
| negsRNA330 | 0.258 | 0.936 | 1.194 | 1794539 | 1794739 | - |  |
| negsRNA322 | 0.294 | 0.900 | 1.193 | 1730188 | 1730388 | - |  |
| negsRNA94 | 0.427 | 0.766 | 1.193 | 507977 | 508177 | - |  |
| negsRNA151 | 0.415 | 0.778 | 1.193 | 662491 | 662691 | - |  |
| negsRNA203 | 0.438 | 0.755 | 1.192 | 971407 | 971607 | - |  |
| negsRNA148 | 0.476 | 0.716 | 1.192 | 659937 | 660137 | - |  |
| possRNA134 | 0.552 | 0.639 | 1.191 | 754631 | 754831 | + |  |
| Zms20 | 0.406 | 0.785 | 1.191 | 258560 | 258585 | - |  |
| possRNA233 | 0.372 | 0.819 | 1.191 | 1374585 | 1374785 | + |  |
| possRNA184 | 0.425 | 0.766 | 1.191 | 1108914 | 1109114 | + |  |
| negsRNA127 | 0.390 | 0.799 | 1.189 | 623826 | 624026 | - |  |
| negsRNA186 | 0.553 | 0.630 | 1.183 | 931842 | 932042 | - |  |
| possRNA311 | 0.667 | 0.512 | 1.179 | 1795659 | 1795859 | + |  |
| possRNA34 | 0.272 | 0.907 | 1.179 | 201393 | 201593 | + |  |
| negsRNA351 | 0.512 | 0.665 | 1.177 | 1897899 | 1898099 | - | tRNA |
| negsRNA291 | 0.399 | 0.777 | 1.176 | 1564869 | 1565069 | - |  |
| negsRNA152 | 0.506 | 0.669 | 1.175 | 663279 | 663479 | - |  |
| possRNA8 | 0.506 | 0.668 | 1.173 | 50502 | 50702 | + |  |
| possRNA275 | 0.438 | 0.733 | 1.171 | 1644313 | 1644513 | + |  |
| negsRNA125 | 0.332 | 0.837 | 1.169 | 617056 | 617256 | - |  |
| possRNA197 | 0.529 | 0.632 | 1.161 | 1198122 | 1198322 | + |  |
| negsRNA80 | 0.414 | 0.746 | 1.159 | 406877 | 407077 | - |  |
| negsRNA228 | 0.308 | 0.850 | 1.158 | 1129317 | 1129517 | - | tRNA |
| negsRNA280 | 0.472 | 0.685 | 1.157 | 1529362 | 1529562 | - |  |
| possRNA237 | 0.446 | 0.710 | 1.156 | 1387232 | 1387432 | + |  |
| possRNA115 | 0.490 | 0.662 | 1.152 | 721549 | 721749 | + | tRNA |
| negsRNA42 | 0.518 | 0.634 | 1.152 | 233946 | 234146 | - |  |
| negsRNA222 | 0.374 | 0.777 | 1.151 | 1108929 | 1109129 | - |  |
| negsRNA40 | 0.447 | 0.704 | 1.151 | 232793 | 232993 | - |  |
| negsRNA126 | 0.150 | 1.000 | 1.150 | 622862 | 623062 | - |  |
| possRNA276 | 0.325 | 0.824 | 1.149 | 1649585 | 1649785 | + |  |
| possRNA120 | 0.534 | 0.614 | 1.148 | 739784 | 739984 | + |  |
| possRNA123 | 0.531 | 0.616 | 1.147 | 745105 | 745305 | + |  |
| negsRNA71 | 0.384 | 0.759 | 1.144 | 372475 | 372675 | - |  |
| negsRNA182 | 0.277 | 0.866 | 1.143 | 875405 | 875605 | - |  |
| possRNA52 | 0.324 | 0.818 | 1.142 | 316085 | 316285 | + |  |
| negsRNA87 | 0.336 | 0.806 | 1.142 | 423345 | 423545 | - |  |
| possRNA189 | 0.484 | 0.658 | 1.141 | 1159634 | 1159834 | + |  |
| negsRNA47 | 0.465 | 0.675 | 1.140 | 235839 | 236039 | - |  |
| negsRNA39 | 0.471 | 0.669 | 1.140 | 232625 | 232825 | - |  |
| possRNA306 | 0.397 | 0.741 | 1.138 | 1776648 | 1776848 | + |  |
| possRNA215 | 0.262 | 0.876 | 1.138 | 1300086 | 1300286 | + |  |
| negsRNA307 | 0.325 | 0.812 | 1.136 | 1625185 | 1625385 | - |  |
| negsRNA129 | 0.221 | 0.915 | 1.136 | 626258 | 626458 | - |  |
| negsRNA82 | 0.469 | 0.667 | 1.136 | 407313 | 407513 | - |  |
| possRNA251 | 0.262 | 0.874 | 1.136 | 1503319 | 1503519 | + |  |
| negsRNA299 | 0.634 | 0.500 | 1.134 | 1612712 | 1612912 | - | tRNA |
| possRNA104 | 0.324 | 0.809 | 1.133 | 622860 | 623060 | + |  |
| negsRNA77 | 0.423 | 0.707 | 1.130 | 405898 | 406098 | - |  |
| negsRNA109 | 0.445 | 0.684 | 1.129 | 522298 | 522498 | - |  |
| possRNA108 | 0.484 | 0.645 | 1.129 | 650998 | 651198 | + |  |
| possRNA65 | 0.255 | 0.874 | 1.129 | 432691 | 432891 | + |  |
| negsRNA41 | 0.417 | 0.708 | 1.125 | 233483 | 233683 | - |  |
| negsRNA32 | 0.503 | 0.619 | 1.121 | 215346 | 215546 | - |  |
| possRNA42 | 0.202 | 0.917 | 1.120 | 241529 | 241729 | + |  |
| negsRNA68 | 0.425 | 0.694 | 1.120 | 370128 | 370328 | - |  |
| possRNA292 | 0.397 | 0.723 | 1.119 | 1742462 | 1742662 | + |  |
| possRNA148 | 0.293 | 0.824 | 1.117 | 932589 | 932789 | + |  |
| negsRNA73 | 0.406 | 0.710 | 1.116 | 374000 | 374200 | - |  |
| negsRNA196 | 0.195 | 0.922 | 1.116 | 966231 | 966431 | - |  |
| negsRNA174 | 0.459 | 0.656 | 1.115 | 749579 | 749861 | - |  |
| possRNA57 | 0.425 | 0.690 | 1.114 | 380353 | 380553 | + | tRNA |
| negsRNA262 | 0.347 | 0.767 | 1.114 | 1427452 | 1427652 | - |  |
| negsRNA2 | 0.369 | 0.745 | 1.114 | 17599 | 17799 | - |  |
| possRNA182 | 0.211 | 0.902 | 1.113 | 1073792 | 1074238 | + |  |
| possRNA111 | 0.460 | 0.653 | 1.113 | 656727 | 656927 | + |  |
| negsRNA35 | 0.411 | 0.700 | 1.111 | 232160 | 232360 | - |  |
| negsRNA58 | 0.374 | 0.732 | 1.107 | 285678 | 285878 | - |  |
| negsRNA36 | 0.460 | 0.646 | 1.106 | 232262 | 232462 | - |  |
| possRNA22 | 0.291 | 0.814 | 1.105 | 151478 | 151678 | + |  |
| Zms18 | 0.351 | 0.751 | 1.101 | 1901164 | 1901303 | - |  |
| possRNA243 | 0.241 | 0.852 | 1.094 | 1423947 | 1424147 | + |  |
| negsRNA239 | 0.324 | 0.767 | 1.092 | 1233917 | 1234117 | - | tRNA |
| possRNA17 | 0.311 | 0.778 | 1.089 | 149886 | 150086 | + |  |
| possRNA96 | 0.371 | 0.717 | 1.088 | 611743 | 611943 | + |  |
| negsRNA213 | 0.371 | 0.716 | 1.087 | 1046455 | 1046655 | - |  |
| negsRNA210 | 0.159 | 0.928 | 1.087 | 1012320 | 1012520 | - |  |
| negsRNA177 | 0.389 | 0.696 | 1.084 | 750062 | 750262 | - |  |
| possRNA319 | 0.419 | 0.663 | 1.082 | 1810071 | 1810271 | + |  |
| negsRNA26 | 0.233 | 0.848 | 1.081 | 182542 | 182742 | - |  |
| possRNA202 | 0.261 | 0.817 | 1.079 | 1233918 | 1234118 | + | tRNA |
| possRNA70 | 0.215 | 0.859 | 1.074 | 444071 | 444271 | + | tRNA |
| negsRNA117 | 0.271 | 0.802 | 1.073 | 540346 | 540546 | - | alpha_tmRNA |
| negsRNA282 | 0.322 | 0.749 | 1.071 | 1540719 | 1540919 | - |  |
| negsRNA69 | 0.374 | 0.697 | 1.071 | 371467 | 371667 | - |  |
| possRNA132 | 0.438 | 0.631 | 1.069 | 749711 | 749911 | + |  |
| possRNA277 | 0.395 | 0.673 | 1.068 | 1649865 | 1650065 | + |  |
| negsRNA16 | 0.175 | 0.893 | 1.068 | 149991 | 150390 | - |  |
| negsRNA135 | 0.146 | 0.920 | 1.066 | 650975 | 651175 | - |  |
| possRNA73 | 0.359 | 0.706 | 1.065 | 467597 | 467797 | + |  |
| possRNA295 | 0.368 | 0.697 | 1.065 | 1749843 | 1750043 | + |  |
| possRNA147 | 0.257 | 0.808 | 1.065 | 920775 | 920975 | + | tRNA |
| negsRNA171 | 0.215 | 0.849 | 1.064 | 745984 | 746184 | - |  |
| possRNA69 | 0.183 | 0.881 | 1.064 | 438262 | 438462 | + |  |
| possRNA241 | 0.288 | 0.775 | 1.064 | 1416925 | 1417125 | + |  |
| possRNA235 | 0.262 | 0.800 | 1.063 | 1386307 | 1386507 | + |  |
| negsRNA356 | 0.305 | 0.757 | 1.062 | 1915168 | 1915408 | - | tRNA |
| possRNA61 | 0.368 | 0.693 | 1.062 | 405203 | 405403 | + |  |
| negsRNA79 | 0.331 | 0.729 | 1.060 | 406366 | 406676 | - |  |
| possRNA18 | 0.235 | 0.823 | 1.058 | 150084 | 150317 | + |  |
| negsRNA303 | 0.085 | 0.972 | 1.057 | 1618058 | 1618258 | - |  |
| negsRNA90 | 0.245 | 0.812 | 1.056 | 444069 | 444269 | - | tRNA |
| possRNA301 | 0.295 | 0.759 | 1.054 | 1767373 | 1767573 | + |  |
| negsRNA100 | 0.176 | 0.877 | 1.053 | 516085 | 516285 | - |  |
| possRNA302 | 0.310 | 0.742 | 1.052 | 1767558 | 1767758 | + |  |
| possRNA39 | 0.236 | 0.816 | 1.051 | 214507 | 214707 | + |  |
| negsRNA240 | 0.251 | 0.799 | 1.050 | 1241976 | 1242176 | - |  |
| negsRNA238 | 0.406 | 0.644 | 1.049 | 1230966 | 1231166 | - | tRNA |
| possRNA171 | 0.235 | 0.814 | 1.049 | 1010914 | 1011114 | + |  |
| possRNA165 | 0.256 | 0.791 | 1.047 | 1007251 | 1007451 | + |  |
| negsRNA241 | 0.277 | 0.770 | 1.047 | 1242258 | 1242458 | - |  |
| possRNA125 | 0.336 | 0.710 | 1.047 | 745885 | 746085 | + |  |
| possRNA143 | 0.355 | 0.691 | 1.046 | 912484 | 912684 | + |  |
| possRNA163 | 0.404 | 0.642 | 1.046 | 1006630 | 1006830 | + |  |
| possRNA67 | 0.260 | 0.784 | 1.043 | 437959 | 438159 | + |  |
| possRNA166 | 0.269 | 0.774 | 1.043 | 1007454 | 1007724 | + |  |
| negsRNA249 | 0.232 | 0.810 | 1.042 | 1356149 | 1356349 | - | tRNA |
| negsRNA3 | 0.360 | 0.681 | 1.041 | 17746 | 17946 | - |  |
| possRNA30 | 0.232 | 0.805 | 1.038 | 171225 | 171425 | + |  |
| negsRNA183 | 0.311 | 0.723 | 1.035 | 888377 | 888577 | - |  |
| negsRNA30 | 0.249 | 0.784 | 1.033 | 214816 | 215016 | - |  |
| negsRNA219 | 0.262 | 0.770 | 1.033 | 1079738 | 1079938 | - | RNaseP_bact_a |
| negsRNA67 | 0.421 | 0.612 | 1.033 | 369742 | 369942 | - |  |
| negsRNA173 | 0.327 | 0.703 | 1.030 | 749382 | 749582 | - |  |
| negsRNA296 | 0.448 | 0.579 | 1.027 | 1607979 | 1608179 | - |  |
| possRNA183 | 0.334 | 0.694 | 1.027 | 1079312 | 1079512 | + |  |
| negsRNA297 | 0.225 | 0.800 | 1.025 | 1608290 | 1608490 | - |  |
| possRNA68 | 0.348 | 0.676 | 1.024 | 438148 | 438348 | + |  |
| negsRNA311 | 0.195 | 0.828 | 1.023 | 1651593 | 1651793 | - |  |
| possRNA131 | 0.368 | 0.654 | 1.022 | 749378 | 749578 | + |  |
| negsRNA121 | 0.444 | 0.575 | 1.019 | 572567 | 572767 | - |  |
| possRNA225 | 0.527 | 0.491 | 1.018 | 1365777 | 1365977 | + |  |
| possRNA230 | 0.266 | 0.750 | 1.017 | 1373752 | 1373952 | + |  |
| possRNA59 | 0.276 | 0.740 | 1.016 | 382348 | 382548 | + |  |
| negsRNA66 | 0.021 | 0.995 | 1.015 | 364110 | 364310 | - |  |
| negsRNA72 | 0.418 | 0.591 | 1.010 | 373375 | 373575 | - |  |
| possRNA282 | 0.226 | 0.782 | 1.008 | 1659565 | 1659765 | + |  |
| negsRNA133 | 0.104 | 0.904 | 1.008 | 638635 | 638835 | - |  |
| possRNA19 | 0.384 | 0.623 | 1.007 | 150718 | 150918 | + |  |
| possRNA91 | 0.157 | 0.850 | 1.007 | 570829 | 571029 | + |  |
| negsRNA33 | 0.165 | 0.841 | 1.006 | 226022 | 226222 | - |  |
| negsRNA187 | 0.182 | 0.824 | 1.006 | 932595 | 932795 | - |  |
| negsRNA332 | 0.421 | 0.584 | 1.005 | 1796353 | 1796553 | - |  |
| negsRNA335 | 0.087 | 0.918 | 1.005 | 1817694 | 1817894 | - | tRNA |
| negsRNA263 | 0.358 | 0.646 | 1.004 | 1438402 | 1438602 | - |  |
| negsRNA46 | 0.439 | 0.565 | 1.004 | 235539 | 235739 | - |  |
| possRNA98 | 0.349 | 0.653 | 1.002 | 622105 | 622305 | + |  |
| possRNA256 | 0.341 | 0.659 | 1.000 | 1530017 | 1530217 | + |  |
| possRNA64 | 0.156 | 0.842 | 0.999 | 432590 | 432790 | + |  |
| possRNA167 | 0.171 | 0.827 | 0.998 | 1007770 | 1007970 | + |  |
| possRNA208 | 0.217 | 0.779 | 0.996 | 1258065 | 1258265 | + |  |
| possRNA152 | 0.173 | 0.823 | 0.996 | 960030 | 960230 | + | tRNA |
| negsRNA230 | 0.194 | 0.802 | 0.996 | 1148762 | 1148962 | - |  |
| possRNA51 | 0.288 | 0.707 | 0.995 | 311125 | 311325 | + |  |
| possRNA278 | 0.400 | 0.594 | 0.995 | 1650023 | 1650223 | + |  |
| possRNA28 | 0.209 | 0.785 | 0.994 | 165909 | 166109 | + |  |
| possRNA9 | 0.274 | 0.718 | 0.992 | 76029 | 76229 | + |  |
| possRNA105 | 0.254 | 0.738 | 0.992 | 640946 | 641146 | + |  |
| negsRNA31 | 0.319 | 0.672 | 0.991 | 215045 | 215245 | - |  |
| possRNA198 | 0.244 | 0.745 | 0.988 | 1205569 | 1205769 | + |  |
| possRNA213 | 0.149 | 0.839 | 0.988 | 1286279 | 1286479 | + |  |
| negsRNA64 | 0.179 | 0.806 | 0.985 | 341953 | 342153 | - |  |
| possRNA239 | 0.226 | 0.759 | 0.985 | 1410405 | 1410605 | + |  |
| possRNA31 | 0.257 | 0.727 | 0.983 | 174749 | 174949 | + | tRNA |
| negsRNA260 | 0.188 | 0.794 | 0.982 | 1423777 | 1423977 | - |  |
| possRNA53 | 0.231 | 0.751 | 0.981 | 317087 | 317287 | + |  |
| negsRNA113 | 0.171 | 0.810 | 0.981 | 531976 | 532176 | - |  |
| possRNA204 | 0.221 | 0.759 | 0.980 | 1241972 | 1242172 | + |  |
| negsRNA268 | 0.212 | 0.766 | 0.979 | 1440841 | 1441041 | - |  |
| negsRNA265 | 0.202 | 0.775 | 0.977 | 1439565 | 1439765 | - |  |
| negsRNA292 | 0.178 | 0.797 | 0.975 | 1578029 | 1578229 | - |  |
| possRNA62 | 0.275 | 0.701 | 0.975 | 408416 | 408616 | + |  |
| negsRNA89 | 0.151 | 0.824 | 0.975 | 438585 | 438785 | - |  |
| negsRNA328 | 0.304 | 0.671 | 0.975 | 1767241 | 1767441 | - |  |
| negsRNA234 | 0.368 | 0.607 | 0.975 | 1173059 | 1173259 | - |  |
| negsRNA227 | 0.140 | 0.835 | 0.975 | 1128237 | 1128437 | - |  |
| negsRNA88 | 0.170 | 0.802 | 0.973 | 438266 | 438466 | - |  |
| negsRNA146 | 0.110 | 0.862 | 0.972 | 653012 | 653212 | - |  |
| negsRNA18 | 0.190 | 0.781 | 0.971 | 151486 | 151686 | - |  |
| negsRNA192 | 0.098 | 0.873 | 0.970 | 961831 | 962031 | - | tRNA |
| negsRNA231 | 0.263 | 0.708 | 0.970 | 1159371 | 1159571 | - |  |
| possRNA224 | 0.296 | 0.673 | 0.969 | 1365657 | 1365857 | + |  |
| possRNA35 | 0.144 | 0.823 | 0.967 | 201562 | 201762 | + |  |
| negsRNA7 | 0.160 | 0.807 | 0.967 | 113463 | 113663 | - | tRNA |
| negsRNA189 | 0.326 | 0.636 | 0.962 | 943125 | 943325 | - |  |
| possRNA54 | 0.254 | 0.708 | 0.961 | 317289 | 317489 | + |  |
| negsRNA154 | 0.161 | 0.798 | 0.959 | 690312 | 690512 | - |  |
| negsRNA270 | 0.097 | 0.862 | 0.959 | 1461365 | 1461565 | - |  |
| possRNA139 | 0.199 | 0.760 | 0.958 | 883540 | 883740 | + |  |
| possRNA44 | 0.342 | 0.615 | 0.957 | 282497 | 282697 | + |  |
| negsRNA272 | 0.113 | 0.842 | 0.955 | 1493434 | 1493634 | - |  |
| negsRNA225 | 0.124 | 0.830 | 0.954 | 1127382 | 1127582 | - |  |
| negsRNA304 | 0.111 | 0.841 | 0.952 | 1623568 | 1623768 | - |  |
| negsRNA327 | 0.391 | 0.560 | 0.951 | 1766006 | 1766206 | - |  |
| negsRNA147 | 0.270 | 0.680 | 0.950 | 656726 | 656926 | - |  |
| negsRNA65 | 0.149 | 0.800 | 0.949 | 358104 | 358304 | - |  |
| negsRNA150 | 0.335 | 0.613 | 0.947 | 661488 | 661688 | - |  |
| negsRNA312 | 0.171 | 0.775 | 0.946 | 1659585 | 1659785 | - |  |
| possRNA240 | 0.199 | 0.747 | 0.945 | 1411117 | 1411317 | + |  |
| possRNA97 | 0.181 | 0.764 | 0.945 | 621989 | 622189 | + |  |
| possRNA15 | 0.266 | 0.679 | 0.945 | 137905 | 138105 | + |  |
| negsRNA98 | 0.248 | 0.697 | 0.944 | 514377 | 514577 | - |  |
| negsRNA143 | 0.123 | 0.821 | 0.944 | 652464 | 652664 | - |  |
| negsRNA81 | 0.286 | 0.657 | 0.943 | 407087 | 407287 | - |  |
| possRNA41 | 0.098 | 0.844 | 0.942 | 241064 | 241264 | + |  |
| negsRNA140 | 0.055 | 0.886 | 0.941 | 651914 | 652114 | - |  |
| possRNA86 | 0.160 | 0.781 | 0.941 | 546023 | 546223 | + |  |
| possRNA250 | 0.176 | 0.765 | 0.941 | 1501165 | 1501365 | + |  |
| possRNA124 | 0.384 | 0.557 | 0.941 | 745360 | 745560 | + |  |
| possRNA20 | 0.397 | 0.543 | 0.940 | 151024 | 151270 | + |  |
| possRNA222 | 0.160 | 0.779 | 0.940 | 1356151 | 1356351 | + | tRNA |
| possRNA129 | 0.150 | 0.790 | 0.939 | 746572 | 746772 | + |  |
| negsRNA279 | 0.173 | 0.761 | 0.934 | 1519893 | 1520093 | - |  |
| possRNA102 | 0.444 | 0.488 | 0.932 | 622581 | 622781 | + |  |
| negsRNA275 | 0.041 | 0.889 | 0.931 | 1515392 | 1515592 | - |  |
| possRNA218 | 0.113 | 0.816 | 0.929 | 1308425 | 1308625 | + |  |
| negsRNA350 | 0.178 | 0.745 | 0.923 | 1882364 | 1882564 | - |  |
| negsRNA271 | 0.185 | 0.736 | 0.921 | 1491280 | 1491480 | - |  |
| negsRNA144 | 0.092 | 0.829 | 0.921 | 652608 | 652808 | - |  |
| possRNA82 | 0.187 | 0.734 | 0.921 | 543664 | 543864 | + |  |
| negsRNA218 | 0.115 | 0.805 | 0.919 | 1079630 | 1079830 | - | RNaseP_bact_a |
| negsRNA211 | 0.048 | 0.869 | 0.917 | 1012662 | 1012862 | - |  |
| negsRNA178 | 0.143 | 0.773 | 0.916 | 770684 | 770884 | - |  |
| possRNA138 | 0.049 | 0.865 | 0.913 | 875402 | 875602 | + |  |
| negsRNA185 | 0.186 | 0.727 | 0.913 | 924475 | 924675 | - |  |
| possRNA33 | 0.211 | 0.701 | 0.913 | 201200 | 201400 | + |  |
| negsRNA344 | 0.148 | 0.765 | 0.913 | 1841090 | 1841290 | - |  |
| negsRNA139 | 0.103 | 0.810 | 0.913 | 651745 | 651945 | - |  |
| negsRNA341 | 0.094 | 0.817 | 0.911 | 1840209 | 1840409 | - |  |
| possRNA45 | 0.339 | 0.571 | 0.910 | 290477 | 290677 | + |  |
| negsRNA285 | 0.231 | 0.679 | 0.910 | 1546059 | 1546259 | - |  |
| possRNA263 | 0.100 | 0.810 | 0.910 | 1565140 | 1565340 | + |  |
| negsRNA145 | 0.229 | 0.679 | 0.908 | 652834 | 653034 | - |  |
| negsRNA120 | 0.239 | 0.668 | 0.906 | 570640 | 570840 | - | tRNA |
| possRNA90 | 0.158 | 0.748 | 0.906 | 570643 | 570843 | + | tRNA |
| negsRNA337 | 0.002 | 0.904 | 0.906 | 1821273 | 1821473 | - |  |
| negsRNA60 | 0.178 | 0.727 | 0.905 | 297696 | 297896 | - |  |
| possRNA279 | 0.203 | 0.701 | 0.904 | 1654267 | 1654467 | + |  |
| possRNA126 | 0.087 | 0.817 | 0.904 | 745980 | 746180 | + |  |
| possRNA135 | 0.173 | 0.731 | 0.903 | 770684 | 770884 | + |  |
| negsRNA293 | 0.154 | 0.749 | 0.903 | 1580873 | 1581073 | - |  |
| possRNA246 | 0.028 | 0.875 | 0.903 | 1465845 | 1466045 | + | tRNA |
| possRNA158 | 0.137 | 0.765 | 0.901 | 971248 | 971448 | + |  |
| possRNA37 | 0.145 | 0.754 | 0.900 | 202039 | 202239 | + |  |
| negsRNA103 | 0.195 | 0.704 | 0.899 | 519420 | 519620 | - |  |
| negsRNA110 | 0.186 | 0.713 | 0.899 | 524397 | 524597 | - |  |
| possRNA205 | 0.035 | 0.864 | 0.899 | 1242256 | 1242456 | + |  |
| negsRNA118 | 0.231 | 0.667 | 0.898 | 554108 | 554308 | - |  |
| negsRNA172 | 0.255 | 0.643 | 0.898 | 747033 | 747233 | - |  |
| negsRNA107 | 0.106 | 0.791 | 0.897 | 521169 | 521369 | - |  |
| negsRNA96 | 0.062 | 0.835 | 0.896 | 513093 | 513293 | - |  |
| possRNA264 | 0.345 | 0.551 | 0.896 | 1565491 | 1565691 | + |  |
| negsRNA316 | 0.250 | 0.646 | 0.896 | 1706364 | 1706564 | - |  |
| negsRNA300 | 0.245 | 0.649 | 0.894 | 1612965 | 1613165 | - |  |
| possRNA71 | 0.126 | 0.768 | 0.894 | 454879 | 455079 | + |  |
| possRNA25 | 0.196 | 0.698 | 0.894 | 157546 | 157746 | + |  |
| negsRNA214 | 0.184 | 0.710 | 0.894 | 1049909 | 1050109 | - | tRNA |
| possRNA315 | 0.027 | 0.866 | 0.892 | 1797424 | 1797624 | + |  |
| negsRNA252 | 0.039 | 0.852 | 0.891 | 1381201 | 1381401 | - |  |
| possRNA161 | 0.123 | 0.768 | 0.891 | 992290 | 992490 | + |  |
| possRNA145 | 0.214 | 0.676 | 0.890 | 913029 | 913229 | + |  |
| possRNA144 | 0.085 | 0.805 | 0.890 | 912669 | 912869 | + |  |
| negsRNA21 | 0.090 | 0.800 | 0.890 | 171254 | 171454 | - |  |
| possRNA300 | 0.217 | 0.672 | 0.889 | 1767257 | 1767457 | + |  |
| negsRNA290 | 0.217 | 0.672 | 0.888 | 1564694 | 1564894 | - |  |
| possRNA247 | 0.040 | 0.848 | 0.888 | 1478386 | 1478586 | + |  |
| negsRNA216 | 0.157 | 0.731 | 0.888 | 1062394 | 1062594 | - |  |
| possRNA121 | 0.073 | 0.814 | 0.888 | 743518 | 743718 | + | tRNA |
| negsRNA325 | 0.092 | 0.795 | 0.887 | 1748898 | 1749098 | - |  |
| negsRNA181 | 0.191 | 0.695 | 0.886 | 868905 | 869105 | - | Bacteria_small_SRP |
| negsRNA184 | 0.042 | 0.841 | 0.884 | 920781 | 920981 | - | tRNA |
| negsRNA48 | 0.226 | 0.657 | 0.883 | 239216 | 239416 | - |  |
| negsRNA156 | 0.096 | 0.786 | 0.882 | 721548 | 721748 | - | tRNA |
| negsRNA57 | 0.078 | 0.803 | 0.882 | 283268 | 283468 | - |  |
| negsRNA195 | 0.181 | 0.701 | 0.881 | 966027 | 966227 | - |  |
| Zms18 | 0.171 | 0.710 | 0.881 | 1901164 | 1901303 | - |  |
| negsRNA101 | 0.198 | 0.683 | 0.880 | 516784 | 516984 | - |  |
| possRNA32 | 0.033 | 0.847 | 0.880 | 182540 | 182740 | + |  |
| negsRNA323 | 0.068 | 0.811 | 0.880 | 1737440 | 1737640 | - |  |
| negsRNA6 | 0.047 | 0.831 | 0.878 | 96315 | 96515 | - |  |
| negsRNA317 | 0.214 | 0.663 | 0.876 | 1706552 | 1706752 | - |  |
| negsRNA11 | 0.215 | 0.661 | 0.876 | 139674 | 139874 | - |  |
| possRNA130 | 0.091 | 0.783 | 0.873 | 746984 | 747235 | + |  |
| possRNA320 | 0.151 | 0.722 | 0.873 | 1810600 | 1810800 | + |  |
| Zms3 | 0.167 | 0.705 | 0.873 | 512975 | 513761 | - |  |
| negsRNA208 | 0.115 | 0.758 | 0.873 | 1007253 | 1007453 | - |  |
| negsRNA244 | 0.064 | 0.808 | 0.872 | 1294764 | 1294964 | - |  |
| possRNA55 | 0.102 | 0.770 | 0.872 | 317430 | 317630 | + | tRNA |
| negsRNA336 | 0.135 | 0.737 | 0.872 | 1818796 | 1818996 | - |  |
| negsRNA29 | 0.060 | 0.812 | 0.872 | 214508 | 214708 | - |  |
| possRNA26 | 0.098 | 0.773 | 0.871 | 157708 | 157908 | + |  |
| negsRNA346 | 0.098 | 0.773 | 0.871 | 1841407 | 1841607 | - |  |
| negsRNA17 | 0.153 | 0.718 | 0.871 | 150656 | 150856 | - |  |
| negsRNA104 | 0.197 | 0.673 | 0.870 | 520486 | 520686 | - |  |
| possRNA149 | 0.153 | 0.716 | 0.869 | 942931 | 943131 | + |  |
| negsRNA205 | 0.088 | 0.779 | 0.867 | 1006356 | 1006556 | - |  |
| negsRNA179 | 0.066 | 0.798 | 0.865 | 841062 | 841262 | - |  |
| Zms24 | 0.226 | 0.639 | 0.864 | 1607580 | 1607625 | - |  |
| possRNA296 | 0.034 | 0.830 | 0.864 | 1755843 | 1756043 | + |  |
| possRNA48 | 0.173 | 0.688 | 0.862 | 290919 | 291119 | + |  |
| negsRNA284 | 0.197 | 0.664 | 0.862 | 1541419 | 1541619 | - |  |
| negsRNA278 | 0.119 | 0.742 | 0.862 | 1515916 | 1516116 | - |  |
| negsRNA22 | 0.127 | 0.734 | 0.862 | 171426 | 171626 | - |  |
| possRNA179 | 0.124 | 0.735 | 0.859 | 1056277 | 1056477 | + | tRNA |
| possRNA16 | 0.115 | 0.744 | 0.858 | 138778 | 138978 | + |  |
| negsRNA226 | 0.133 | 0.726 | 0.858 | 1127523 | 1127723 | - |  |
| negsRNA52 | 0.222 | 0.636 | 0.858 | 242307 | 242507 | - |  |
| negsRNA53 | 0.066 | 0.792 | 0.858 | 258486 | 258686 | - |  |
| negsRNA267 | 0.039 | 0.818 | 0.857 | 1440413 | 1440613 | - |  |
| possRNA187 | 0.232 | 0.624 | 0.856 | 1138695 | 1138895 | + | tRNA |
| possRNA322 | 0.044 | 0.812 | 0.856 | 1817696 | 1817896 | + | tRNA |
| negsRNA159 | 0.124 | 0.731 | 0.855 | 725581 | 725781 | - |  |
| negsRNA246 | 0.085 | 0.769 | 0.854 | 1345927 | 1346127 | - |  |
| possRNA211 | 0.132 | 0.722 | 0.854 | 1262556 | 1262756 | + |  |
| negsRNA84 | 0.065 | 0.790 | 0.854 | 409918 | 410118 | - |  |
| possRNA11 | 0.219 | 0.634 | 0.853 | 113492 | 113692 | + | tRNA |
| negsRNA318 | 0.128 | 0.723 | 0.852 | 1707214 | 1707414 | - |  |
| negsRNA247 | 0.073 | 0.778 | 0.851 | 1350494 | 1350694 | - |  |
| possRNA60 | 0.210 | 0.641 | 0.851 | 382545 | 382745 | + |  |
| negsRNA223 | 0.010 | 0.840 | 0.850 | 1127003 | 1127203 | - |  |
| possRNA156 | 0.181 | 0.669 | 0.849 | 965723 | 965923 | + |  |
| negsRNA194 | 0.026 | 0.822 | 0.848 | 965727 | 965927 | - |  |
| negsRNA61 | 0.193 | 0.655 | 0.848 | 311129 | 311329 | - |  |
| negsRNA245 | 0.173 | 0.675 | 0.848 | 1334927 | 1335127 | - |  |
| possRNA74 | 0.036 | 0.812 | 0.847 | 475555 | 475755 | + |  |
| negsRNA334 | 0.069 | 0.778 | 0.847 | 1801377 | 1801577 | - |  |
| possRNA85 | 0.121 | 0.724 | 0.846 | 545843 | 546043 | + |  |
| possRNA175 | 0.049 | 0.797 | 0.846 | 1047722 | 1047922 | + |  |
| possRNA234 | 0.089 | 0.757 | 0.845 | 1384168 | 1384368 | + | tRNA |
| possRNA168 | 0.108 | 0.736 | 0.844 | 1010223 | 1010423 | + |  |
| possRNA253 | 0.125 | 0.718 | 0.843 | 1513870 | 1514070 | + |  |
| negsRNA112 | 0.012 | 0.831 | 0.843 | 527634 | 527834 | - |  |
| possRNA214 | 0.077 | 0.765 | 0.842 | 1294551 | 1294751 | + |  |
| negsRNA347 | 0.112 | 0.727 | 0.840 | 1841788 | 1841988 | - |  |
| negsRNA206 | 0.146 | 0.693 | 0.839 | 1006586 | 1006786 | - |  |
| possRNA203 | 0.215 | 0.624 | 0.839 | 1235592 | 1235792 | + |  |
| negsRNA51 | 0.148 | 0.690 | 0.839 | 242159 | 242359 | - |  |
| negsRNA235 | 0.116 | 0.722 | 0.838 | 1205568 | 1205768 | - |  |
| possRNA281 | 0.159 | 0.679 | 0.838 | 1659219 | 1659419 | + |  |
| possRNA257 | 0.037 | 0.798 | 0.836 | 1540857 | 1541057 | + |  |
| negsRNA142 | 0.028 | 0.808 | 0.836 | 652238 | 652438 | - |  |
| possRNA317 | 0.144 | 0.691 | 0.835 | 1797882 | 1798082 | + |  |
| negsRNA102 | 0.069 | 0.765 | 0.835 | 518107 | 518307 | - |  |
| possRNA136 | 0.035 | 0.799 | 0.834 | 818165 | 818365 | + |  |
| possRNA133 | 0.139 | 0.695 | 0.834 | 750061 | 750261 | + |  |
| negsRNA188 | 0.029 | 0.804 | 0.833 | 942939 | 943139 | - |  |
| possRNA83 | 0.255 | 0.578 | 0.833 | 544428 | 544628 | + |  |
| negsRNA161 | 0.006 | 0.826 | 0.832 | 726699 | 726899 | - |  |
| negsRNA169 | 0.034 | 0.797 | 0.830 | 743510 | 743710 | - | tRNA |
| possRNA194 | 0.111 | 0.718 | 0.829 | 1173410 | 1173610 | + |  |
| possRNA191 | 0.181 | 0.647 | 0.828 | 1162379 | 1162579 | + |  |
| negsRNA59 | 0.139 | 0.688 | 0.827 | 291611 | 291811 | - |  |
| negsRNA200 | 0.117 | 0.709 | 0.826 | 967606 | 967806 | - |  |
| negsRNA221 | 0.123 | 0.702 | 0.825 | 1086366 | 1086566 | - |  |
| possRNA79 | 0.061 | 0.763 | 0.824 | 540348 | 540548 | + | alpha_tmRNA |
| possRNA286 | 0.089 | 0.736 | 0.824 | 1700110 | 1700310 | + |  |
| possRNA221 | 0.097 | 0.727 | 0.824 | 1351035 | 1351235 | + |  |
| negsRNA277 | 0.111 | 0.711 | 0.822 | 1515751 | 1515951 | - |  |
| negsRNA345 | 0.019 | 0.803 | 0.822 | 1841163 | 1841363 | - |  |
| negsRNA273 | 0.176 | 0.646 | 0.822 | 1510277 | 1510477 | - |  |
| negsRNA237 | 0.057 | 0.765 | 0.822 | 1223858 | 1224058 | - |  |
| possRNA107 | 0.022 | 0.799 | 0.821 | 644647 | 644847 | + |  |
| possRNA47 | 0.078 | 0.742 | 0.821 | 290786 | 290986 | + |  |
| negsRNA124 | 0.172 | 0.648 | 0.820 | 597672 | 597872 | - |  |
| Zms4 | 0.101 | 0.719 | 0.820 | 1350990 | 1351016 | - |  |
| possRNA106 | 0.083 | 0.736 | 0.819 | 641164 | 641364 | + |  |
| negsRNA158 | 0.132 | 0.687 | 0.819 | 725301 | 725501 | - |  |
| possRNA255 | 0.208 | 0.610 | 0.817 | 1529839 | 1530039 | + |  |
| negsRNA5 | 0.019 | 0.798 | 0.817 | 89901 | 90101 | - |  |
| negsRNA95 | 0.039 | 0.778 | 0.816 | 511442 | 511642 | - |  |
| possRNA36 | 0.082 | 0.734 | 0.816 | 201821 | 202021 | + |  |
| possRNA269 | 0.164 | 0.652 | 0.816 | 1612879 | 1613079 | + | 5S_rRNA |
| possRNA249 | 0.172 | 0.643 | 0.815 | 1498519 | 1498719 | + |  |
| negsRNA191 | 0.006 | 0.808 | 0.814 | 960034 | 960234 | - | tRNA |
| negsRNA352 | 0.203 | 0.610 | 0.813 | 1898118 | 1898318 | - |  |
| negsRNA308 | 0.013 | 0.800 | 0.813 | 1627428 | 1627628 | - |  |
| negsRNA320 | 0.100 | 0.713 | 0.813 | 1729243 | 1729443 | - | serC |
| possRNA270 | 0.169 | 0.643 | 0.813 | 1615941 | 1616141 | + |  |
| negsRNA128 | 0.082 | 0.730 | 0.812 | 625776 | 625976 | - |  |
| negsRNA329 | 0.050 | 0.763 | 0.812 | 1776654 | 1776854 | - |  |
| possRNA24 | 0.286 | 0.526 | 0.812 | 157313 | 157513 | + |  |
| negsRNA199 | 0.081 | 0.730 | 0.811 | 967327 | 967527 | - |  |
| negsRNA340 | 0.178 | 0.633 | 0.811 | 1837528 | 1837728 | - | tRNA |
| possRNA109 | 0.174 | 0.636 | 0.810 | 651185 | 651385 | + |  |
| possRNA84 | 0.115 | 0.695 | 0.810 | 544564 | 544764 | + |  |
| possRNA248 | 0.136 | 0.673 | 0.810 | 1491273 | 1491473 | + |  |
| negsRNA83 | 0.115 | 0.695 | 0.809 | 409110 | 409310 | - |  |
| possRNA210 | 0.089 | 0.720 | 0.809 | 1262471 | 1262671 | + |  |
| possRNA299 | 0.079 | 0.730 | 0.809 | 1762790 | 1762990 | + |  |
| possRNA58 | 0.098 | 0.711 | 0.808 | 381204 | 381404 | + |  |
| negsRNA339 | 0.068 | 0.739 | 0.808 | 1834268 | 1834468 | - |  |
| possRNA190 | 0.150 | 0.657 | 0.807 | 1162034 | 1162234 | + |  |
| negsRNA224 | 0.024 | 0.783 | 0.806 | 1127193 | 1127393 | - |  |
| negsRNA207 | 0.135 | 0.671 | 0.806 | 1007013 | 1007213 | - |  |
| possRNA160 | 0.164 | 0.642 | 0.806 | 991974 | 992174 | + | tRNA |
| possRNA290 | 0.090 | 0.715 | 0.806 | 1706254 | 1706454 | + |  |
| possRNA287 | 0.134 | 0.672 | 0.806 | 1700963 | 1701163 | + |  |
| negsRNA197 | 0.009 | 0.797 | 0.805 | 966483 | 966683 | - |  |
| negsRNA243 | 0.017 | 0.787 | 0.804 | 1260334 | 1260534 | - |  |
| possRNA6 | 0.117 | 0.687 | 0.804 | 40182 | 40382 | + |  |
| negsRNA309 | 0.051 | 0.752 | 0.804 | 1633730 | 1633930 | - |  |
| possRNA209 | 0.213 | 0.590 | 0.802 | 1259689 | 1259889 | + |  |
| Zms16 | 0.142 | 0.661 | 0.802 | 868916 | 869028 | - |  |
| negsRNA86 | 0.088 | 0.714 | 0.802 | 414861 | 415061 | - |  |
| possRNA316 | 0.000 | 0.801 | 0.802 | 1797567 | 1797767 | + |  |
| negsRNA283 | 0.035 | 0.765 | 0.800 | 1541151 | 1541351 | - |  |
| possRNA258 | 0.011 | 0.789 | 0.800 | 1541103 | 1541303 | + |  |
| possRNA89 | 0.108 | 0.691 | 0.799 | 559391 | 559591 | + |  |
| negsRNA114 | 0.009 | 0.790 | 0.799 | 532647 | 532847 | - |  |
| possRNA119 | 0.092 | 0.705 | 0.797 | 737566 | 737766 | + |  |
| negsRNA342 | 0.019 | 0.777 | 0.797 | 1840375 | 1840575 | - |  |
| possRNA150 | 0.038 | 0.759 | 0.796 | 946685 | 946885 | + |  |
| possRNA95 | 0.169 | 0.627 | 0.796 | 610759 | 610959 | + |  |
| negsRNA315 | 0.227 | 0.569 | 0.796 | 1706259 | 1706459 | - |  |
| negsRNA25 | 0.052 | 0.744 | 0.795 | 174752 | 174952 | - | tRNA |
| negsRNA91 | 0.078 | 0.715 | 0.794 | 444502 | 444702 | - |  |
| possRNA298 | 0.024 | 0.768 | 0.791 | 1761119 | 1761319 | + |  |
| negsRNA49 | 0.020 | 0.770 | 0.790 | 239504 | 239704 | - |  |
| possRNA266 | 0.035 | 0.755 | 0.790 | 1598120 | 1598320 | + |  |
| possRNA186 | 0.003 | 0.786 | 0.789 | 1129316 | 1129516 | + | tRNA |
| possRNA337 | 0.012 | 0.777 | 0.789 | 1915194 | 1915406 | + | tRNA |
| possRNA314 | 0.010 | 0.778 | 0.788 | 1797325 | 1797525 | + |  |
| possRNA244 | 0.014 | 0.774 | 0.788 | 1457000 | 1457200 | + |  |
| negsRNA264 | 0.029 | 0.760 | 0.788 | 1438991 | 1439191 | - |  |
| negsRNA266 | 0.074 | 0.714 | 0.788 | 1439946 | 1440146 | - |  |
| negsRNA132 | 0.019 | 0.767 | 0.786 | 637598 | 637798 | - |  |
| possRNA220 | 0.142 | 0.643 | 0.785 | 1334922 | 1335122 | + |  |
| negsRNA229 | 0.030 | 0.754 | 0.784 | 1138695 | 1138895 | - | tRNA |
| possRNA118 | 0.085 | 0.697 | 0.781 | 737450 | 737650 | + |  |
| negsRNA141 | 0.045 | 0.736 | 0.781 | 652058 | 652258 | - |  |
| possRNA5 | 0.016 | 0.765 | 0.781 | 39924 | 40124 | + |  |
| negsRNA255 | 0.020 | 0.761 | 0.781 | 1385936 | 1386136 | - |  |
| negsRNA15 | 0.036 | 0.744 | 0.780 | 148744 | 148944 | - |  |
| negsRNA10 | 0.044 | 0.736 | 0.779 | 138785 | 138985 | - |  |
| possRNA201 | 0.171 | 0.607 | 0.778 | 1231931 | 1232131 | + |  |
| possRNA46 | 0.149 | 0.630 | 0.778 | 290597 | 290797 | + |  |
| possRNA188 | 0.117 | 0.660 | 0.777 | 1148532 | 1148732 | + |  |
| possRNA185 | 0.005 | 0.773 | 0.777 | 1126914 | 1127114 | + |  |
| negsRNA242 | 0.041 | 0.736 | 0.777 | 1253364 | 1253564 | - | tRNA |
| negsRNA306 | 0.020 | 0.756 | 0.777 | 1624021 | 1624221 | - |  |
| possRNA321 | 0.078 | 0.698 | 0.776 | 1815298 | 1815498 | + |  |
| possRNA116 | 0.000 | 0.776 | 0.776 | 721725 | 721925 | + |  |
| negsRNA276 | 0.090 | 0.685 | 0.776 | 1515612 | 1515812 | - |  |
| possRNA38 | 0.036 | 0.740 | 0.776 | 214289 | 214489 | + |  |
| possRNA178 | 0.050 | 0.725 | 0.775 | 1049910 | 1050110 | + | tRNA |
| possRNA177 | 0.053 | 0.722 | 0.775 | 1048052 | 1048252 | + |  |
| possRNA78 | 0.045 | 0.730 | 0.775 | 540147 | 540353 | + | alpha_tmRNA |
| negsRNA50 | 0.020 | 0.753 | 0.773 | 241064 | 241264 | - |  |
| possRNA29 | 0.162 | 0.612 | 0.773 | 166783 | 166983 | + |  |
| negsRNA157 | 0.115 | 0.655 | 0.770 | 722823 | 723023 | - |  |
| negsRNA134 | 0.078 | 0.692 | 0.770 | 640760 | 640960 | - |  |
| negsRNA4 | 0.095 | 0.674 | 0.770 | 18274 | 18474 | - |  |
| possRNA113 | 0.136 | 0.633 | 0.769 | 690133 | 690333 | + |  |
| possRNA280 | 0.096 | 0.671 | 0.767 | 1654506 | 1654706 | + |  |
| possRNA49 | 0.181 | 0.586 | 0.767 | 297500 | 297700 | + |  |
| negsRNA123 | 0.110 | 0.656 | 0.766 | 596826 | 597026 | - |  |
| possRNA288 | 0.010 | 0.756 | 0.765 | 1701138 | 1701338 | + |  |
| Zms10 | 0.125 | 0.639 | 0.764 | 39493 | 39517 | - |  |
| negsRNA201 | 0.221 | 0.543 | 0.764 | 971155 | 971355 | - |  |
| possRNA272 | 0.106 | 0.656 | 0.762 | 1623456 | 1623656 | + |  |
| negsRNA254 | 0.075 | 0.686 | 0.761 | 1385814 | 1386014 | - |  |
| possRNA1 | 0.076 | 0.686 | 0.761 | 11555 | 11755 | + |  |
| possRNA236 | 0.031 | 0.731 | 0.761 | 1387131 | 1387331 | + |  |
| negsRNA160 | 0.042 | 0.718 | 0.760 | 726462 | 726662 | - |  |
| possRNA140 | 0.063 | 0.697 | 0.760 | 884868 | 885068 | + |  |
| negsRNA166 | 0.034 | 0.726 | 0.760 | 730212 | 730412 | - |  |
| possRNA99 | 0.176 | 0.583 | 0.759 | 622227 | 622427 | + |  |
| possRNA87 | 0.032 | 0.727 | 0.759 | 553449 | 553649 | + |  |
| possRNA66 | 0.017 | 0.741 | 0.759 | 432989 | 433189 | + |  |
| possRNA206 | 0.012 | 0.747 | 0.758 | 1246987 | 1247187 | + |  |
| negsRNA23 | 0.035 | 0.723 | 0.758 | 171919 | 172119 | - |  |
| possRNA242 | 0.149 | 0.609 | 0.758 | 1417461 | 1417661 | + |  |
| possRNA291 | 0.035 | 0.722 | 0.756 | 1737430 | 1737630 | + |  |
| negsRNA8 | 0.023 | 0.733 | 0.756 | 125299 | 125499 | - |  |
| possRNA180 | 0.025 | 0.731 | 0.756 | 1062394 | 1062594 | + |  |
| negsRNA162 | 0.011 | 0.745 | 0.756 | 726830 | 727030 | - |  |
| negsRNA289 | 0.166 | 0.589 | 0.756 | 1563964 | 1564164 | - |  |
| possRNA271 | 0.040 | 0.715 | 0.754 | 1616161 | 1616361 | + | tRNA |
| negsRNA131 | 0.109 | 0.644 | 0.753 | 630457 | 630657 | - |  |
| possRNA313 | 0.025 | 0.728 | 0.753 | 1797134 | 1797334 | + |  |
| possRNA92 | 0.034 | 0.719 | 0.753 | 588738 | 588938 | + |  |
| possRNA40 | 0.013 | 0.738 | 0.750 | 227399 | 227599 | + | tRNA |
| negsRNA319 | 0.041 | 0.710 | 0.750 | 1707452 | 1707652 | - |  |
| possRNA285 | 0.040 | 0.710 | 0.750 | 1692292 | 1692492 | + | tRNA |
| possRNA169 | 0.145 | 0.605 | 0.749 | 1010475 | 1010675 | + |  |
| negsRNA108 | 0.184 | 0.564 | 0.748 | 521863 | 522063 | - |  |
| possRNA164 | 0.087 | 0.660 | 0.747 | 1007013 | 1007213 | + |  |
| possRNA217 | 0.096 | 0.651 | 0.747 | 1303263 | 1303463 | + |  |
| negsRNA168 | 0.132 | 0.614 | 0.746 | 739749 | 739949 | - |  |
| negsRNA153 | 0.100 | 0.646 | 0.746 | 679162 | 679362 | - |  |
| possRNA200 | 0.110 | 0.635 | 0.745 | 1230966 | 1231166 | + | tRNA |
| negsRNA343 | 0.116 | 0.628 | 0.744 | 1840872 | 1841072 | - |  |
| negsRNA204 | 0.054 | 0.690 | 0.744 | 991980 | 992180 | - | tRNA |
| negsRNA170 | 0.035 | 0.708 | 0.743 | 745897 | 746097 | - |  |
| possRNA81 | 0.105 | 0.637 | 0.742 | 543552 | 543752 | + |  |
| negsRNA12 | 0.094 | 0.646 | 0.741 | 148033 | 148233 | - |  |
| negsRNA9 | 0.038 | 0.702 | 0.740 | 125513 | 125713 | - |  |
| possRNA310 | 0.013 | 0.727 | 0.740 | 1794539 | 1794739 | + |  |
| possRNA195 | 0.022 | 0.717 | 0.739 | 1173712 | 1173912 | + |  |
| possRNA265 | 0.181 | 0.556 | 0.738 | 1593617 | 1593817 | + | tRNA |
| possRNA93 | 0.053 | 0.684 | 0.737 | 603759 | 603959 | + |  |
| negsRNA215 | 0.000 | 0.735 | 0.736 | 1056277 | 1056477 | - | tRNA |
| possRNA174 | 0.016 | 0.719 | 0.735 | 1040135 | 1040335 | + |  |
| negsRNA105 | 0.056 | 0.677 | 0.734 | 520780 | 520980 | - |  |
| negsRNA1 | 0.000 | 0.731 | 0.731 | 12645 | 12845 | - |  |
| negsRNA295 | 0.038 | 0.691 | 0.729 | 1607559 | 1607759 | - |  |
| negsRNA165 | 0.018 | 0.711 | 0.729 | 729280 | 729480 | - |  |
| possRNA181 | 0.074 | 0.655 | 0.729 | 1073528 | 1073729 | + |  |
| negsRNA288 | 0.043 | 0.685 | 0.728 | 1563639 | 1563839 | - |  |
| possRNA297 | 0.025 | 0.703 | 0.728 | 1760952 | 1761152 | + |  |
| possRNA94 | 0.031 | 0.697 | 0.728 | 610043 | 610243 | + |  |
| negsRNA250 | 0.025 | 0.698 | 0.724 | 1356301 | 1356501 | - |  |
| possRNA27 | 0.097 | 0.626 | 0.723 | 158085 | 158285 | + |  |
| possRNA88 | 0.041 | 0.679 | 0.721 | 554100 | 554300 | + |  |
| negsRNA116 | 0.116 | 0.604 | 0.720 | 540149 | 540350 | - | alpha_tmRNA |
| possRNA227 | 0.024 | 0.694 | 0.718 | 1367576 | 1367776 | + |  |
| possRNA289 | 0.107 | 0.611 | 0.718 | 1702957 | 1703157 | + |  |
| negsRNA149 | 0.019 | 0.697 | 0.717 | 660413 | 660613 | - |  |
| possRNA72 | 0.119 | 0.597 | 0.716 | 462321 | 462521 | + |  |
| negsRNA220 | 0.065 | 0.651 | 0.716 | 1085085 | 1085285 | - |  |
| possRNA142 | 0.025 | 0.690 | 0.715 | 890260 | 890460 | + |  |
| negsRNA28 | 0.029 | 0.685 | 0.715 | 201665 | 201865 | - |  |
| possRNA146 | 0.019 | 0.695 | 0.714 | 913381 | 913581 | + |  |
| negsRNA298 | 0.245 | 0.469 | 0.714 | 1612407 | 1612607 | - |  |
| negsRNA34 | 0.067 | 0.646 | 0.714 | 227404 | 227604 | - | tRNA |
| possRNA303 | 0.087 | 0.626 | 0.713 | 1768189 | 1768389 | + |  |
| negsRNA14 | 0.065 | 0.647 | 0.712 | 148616 | 148816 | - |  |
| possRNA309 | 0.016 | 0.696 | 0.712 | 1793464 | 1793664 | + |  |
| negsRNA62 | 0.089 | 0.620 | 0.709 | 317298 | 317498 | - |  |
| negsRNA27 | 0.087 | 0.622 | 0.709 | 194830 | 195030 | - |  |
| possRNA173 | 0.058 | 0.649 | 0.708 | 1028552 | 1028752 | + | tRNA |
| negsRNA136 | 0.002 | 0.705 | 0.707 | 651188 | 651388 | - |  |
| possRNA170 | 0.021 | 0.685 | 0.706 | 1010651 | 1010851 | + |  |
| possRNA193 | 0.059 | 0.647 | 0.706 | 1173101 | 1173301 | + |  |
| possRNA294 | 0.063 | 0.642 | 0.705 | 1748897 | 1749097 | + |  |
| negsRNA163 | 0.017 | 0.688 | 0.705 | 727282 | 727482 | - |  |
| negsRNA314 | 0.026 | 0.679 | 0.704 | 1667244 | 1667444 | - |  |
| negsRNA258 | 0.009 | 0.695 | 0.704 | 1411142 | 1411342 | - |  |
| possRNA207 | 0.097 | 0.606 | 0.703 | 1253397 | 1253597 | + | tRNA |
| possRNA50 | 0.023 | 0.680 | 0.703 | 297711 | 297911 | + |  |
| possRNA176 | 0.041 | 0.660 | 0.701 | 1047856 | 1048056 | + |  |
| possRNA141 | 0.028 | 0.673 | 0.701 | 884990 | 885190 | + |  |
| negsRNA97 | 0.054 | 0.647 | 0.701 | 514030 | 514230 | - |  |
| possRNA261 | 0.013 | 0.685 | 0.698 | 1564701 | 1564901 | + |  |
| negsRNA93 | 0.018 | 0.679 | 0.698 | 476679 | 476879 | - |  |
| possRNA117 | 0.035 | 0.663 | 0.698 | 725303 | 725503 | + |  |
| possRNA112 | 0.145 | 0.552 | 0.697 | 689899 | 690099 | + |  |
| possRNA4 | 0.027 | 0.668 | 0.696 | 39647 | 39847 | + |  |
| negsRNA20 | 0.089 | 0.606 | 0.695 | 166786 | 166986 | - |  |
| negsRNA321 | 0.026 | 0.668 | 0.694 | 1730104 | 1730304 | - |  |
| possRNA312 | 0.132 | 0.559 | 0.691 | 1796740 | 1796940 | + |  |
| negsRNA167 | 0.011 | 0.680 | 0.691 | 739491 | 739691 | - |  |
| possRNA10 | 0.075 | 0.614 | 0.690 | 110440 | 110640 | + |  |
| possRNA254 | 0.003 | 0.687 | 0.690 | 1515758 | 1515958 | + |  |
| Zms15 | 0.000 | 0.688 | 0.688 | 1666725 | 1666996 | - |  |
| negsRNA55 | 0.017 | 0.671 | 0.687 | 258846 | 259046 | - |  |
| possRNA162 | 0.056 | 0.631 | 0.687 | 993011 | 993211 | + |  |
| possRNA155 | 0.069 | 0.615 | 0.684 | 963018 | 963218 | + | tRNA |
| negsRNA193 | 0.003 | 0.678 | 0.681 | 963020 | 963220 | - | tRNA |
| negsRNA236 | 0.033 | 0.646 | 0.679 | 1209520 | 1209720 | - | tRNA |
| possRNA196 | 0.049 | 0.628 | 0.677 | 1181321 | 1181521 | + |  |
| negsRNA190 | 0.039 | 0.637 | 0.676 | 946695 | 946895 | - |  |
| negsRNA287 | 0.043 | 0.632 | 0.675 | 1562260 | 1562460 | - |  |
| possRNA283 | 0.016 | 0.659 | 0.675 | 1660921 | 1661121 | + |  |
| possRNA219 | 0.021 | 0.653 | 0.675 | 1308624 | 1308824 | + |  |
| negsRNA198 | 0.102 | 0.572 | 0.675 | 966750 | 966950 | - |  |
| possRNA259 | 0.026 | 0.648 | 0.674 | 1541420 | 1541620 | + |  |
| negsRNA106 | 0.126 | 0.548 | 0.674 | 520899 | 521099 | - |  |
| negsRNA257 | 0.029 | 0.645 | 0.674 | 1410468 | 1410668 | - |  |
| possRNA151 | 0.005 | 0.670 | 0.674 | 952182 | 952382 | + |  |
| negsRNA253 | 0.008 | 0.665 | 0.673 | 1384181 | 1384381 | - | tRNA |
| possRNA216 | 0.080 | 0.592 | 0.673 | 1300861 | 1301061 | + |  |
| negsRNA115 | 0.134 | 0.537 | 0.671 | 539757 | 539957 | - |  |
| possRNA56 | 0.014 | 0.654 | 0.668 | 370128 | 370328 | + |  |
| negsRNA259 | 0.052 | 0.616 | 0.668 | 1417457 | 1417657 | - |  |
| negsRNA138 | 0.029 | 0.636 | 0.665 | 651668 | 651868 | - |  |
| possRNA80 | 0.021 | 0.642 | 0.663 | 543320 | 543520 | + |  |
| possRNA192 | 0.021 | 0.642 | 0.663 | 1172804 | 1173004 | + |  |
| possRNA226 | 0.022 | 0.641 | 0.662 | 1367431 | 1367631 | + |  |
| negsRNA305 | 0.024 | 0.637 | 0.661 | 1623938 | 1624138 | - |  |
| negsRNA130 | 0.104 | 0.556 | 0.660 | 628835 | 629035 | - |  |
| negsRNA313 | 0.059 | 0.600 | 0.660 | 1666854 | 1667054 | - |  |
| possRNA23 | 0.066 | 0.592 | 0.659 | 155252 | 155452 | + | TPP |
| negsRNA54 | 0.016 | 0.642 | 0.658 | 258733 | 258933 | - |  |
| negsRNA348 | 0.033 | 0.625 | 0.658 | 1843808 | 1844008 | - |  |
| negsRNA349 | 0.018 | 0.639 | 0.658 | 1843930 | 1844130 | - |  |
| negsRNA13 | 0.015 | 0.642 | 0.656 | 148327 | 148527 | - |  |
| possRNA127 | 0.062 | 0.594 | 0.656 | 746130 | 746330 | + |  |
| possRNA262 | 0.025 | 0.631 | 0.655 | 1564869 | 1565069 | + |  |
| possRNA318 | 0.122 | 0.531 | 0.653 | 1798016 | 1798216 | + |  |
| possRNA199 | 0.059 | 0.594 | 0.653 | 1209484 | 1209684 | + | tRNA |
| negsRNA99 | 0.010 | 0.642 | 0.652 | 514497 | 514697 | - |  |
| negsRNA281 | 0.000 | 0.651 | 0.651 | 1540590 | 1540790 | - |  |
| possRNA110 | 0.072 | 0.578 | 0.650 | 653185 | 653385 | + |  |
| negsRNA155 | 0.003 | 0.646 | 0.649 | 719727 | 719927 | - |  |
| negsRNA24 | 0.025 | 0.621 | 0.645 | 172042 | 172242 | - |  |
| negsRNA269 | 0.099 | 0.544 | 0.643 | 1457288 | 1457488 | - |  |
| negsRNA326 | 0.087 | 0.556 | 0.643 | 1760711 | 1760911 | - |  |
| negsRNA111 | 0.104 | 0.538 | 0.642 | 525889 | 526089 | - |  |
| negsRNA164 | 0.050 | 0.589 | 0.639 | 729096 | 729296 | - |  |
| possRNA228 | 0.071 | 0.563 | 0.634 | 1367782 | 1367982 | + |  |
| possRNA307 | 0.009 | 0.622 | 0.631 | 1781308 | 1781508 | + |  |
| possRNA154 | 0.017 | 0.609 | 0.626 | 962039 | 962239 | + |  |
| possRNA252 | 0.052 | 0.565 | 0.617 | 1511601 | 1511801 | + | tRNA |
| possRNA172 | 0.035 | 0.576 | 0.610 | 1019138 | 1019338 | + |  |
| negsRNA137 | 0.000 | 0.607 | 0.607 | 651370 | 651570 | - |  |
| negsRNA85 | 0.026 | 0.566 | 0.593 | 412818 | 413018 | - |  |
| negsRNA56 | 0.019 | 0.566 | 0.585 | 282499 | 282699 | - |  |
| possRNA153 | 0.027 | 0.551 | 0.578 | 960755 | 960955 | + |  |
| negsRNA274 | 0.009 | 0.565 | 0.574 | 1511601 | 1511801 | - | tRNA |
| possRNA128 | 0.057 | 0.516 | 0.573 | 746277 | 746477 | + |  |
| possRNA63 | 0.037 | 0.532 | 0.570 | 412816 | 413016 | + |  |
| possRNA293 | 0.013 | 0.556 | 0.569 | 1745289 | 1745489 | + |  |
| negsRNA331 | 0.006 | 0.556 | 0.562 | 1794676 | 1794876 | - |  |
| possRNA159 | 0.013 | 0.546 | 0.558 | 977643 | 977843 | + |  |
| negsRNA294 | 0.034 | 0.509 | 0.543 | 1593619 | 1593819 | - | tRNA |
| possRNA268 | 0.014 | 0.435 | 0.449 | 1612707 | 1612907 | + | tRNA |

## Supplementary Table 2.

**Supplementary Table S2. sRNA sequences used for testing in ethanol stress assays.**

| sRNA Name | Sequence (5' to 3') |
| --- | --- |
| Zms1 | AGAAGCGGTTGCCCGTGCGATTGTCGGTTTACCGCATCAATAACCTCTTTTTCGCGATTAAAGGCCGCTGTCTAAGAATAATCCTCGACATTAGGGTGATTTTTGGTATCACCCTTCCGACCAGAAGGTCGGGCGGCCGCCGGTATGTTTTTGACATACGGGAGGAAAGTCCGGGCTCCAAGGAGTAACGGTGCCGGATAACATCCGGCGAGGGTGACCTCAGGGAAAGTGCCACAGAAAACAAACCGCTTTTTACTTCGGTAATAAAGTAAGGATGAAAAGGTGCGGTAAGAGCGCACCGCATTTCCGGTAACGGCAATGGCAAGGTAAACCCCACCGGGAGCAAAACCGAATAGGGATGGCATATGGGCTGGCTCCGCCCCGTCATCCGGGTTGGTTGCTGAAGACCGGAAGCAATTCCGGTTTTAGATGAATGGTCGCCTATCCAGCTTTGCTGGTGGACAGAACCCGGCTTATAGACCTTCTGGCGTATCACCTCTGTTGATTTTCTTTGTCATTTGCCTCTTTTTCCTAAAGAAACTATATTCTTGGTTTTTCCGACTTTGGCCAATTTT |
| Zms2 | ACCAAACCGTAATTGGGGTCGGACCATTATGACCGGCGAAACAACGGGCATTGGGGAAATGGCCTTTATTCCGCAAAATATCGATAAACACACGATAGCGGCCTTCTTTATGCAGGCGCTCAATCGCTTGGCTGAAAATATGTTTGTAATCCAAAGGATGACACTCCTTTTGACGAAAGAGGCTTTTGTGACAGCCTGACTATAAGAAGGAAGAATAGATCCGCTTTTAACCCTGTCTTAAAGATCAGACTAGGGAAAAAACGAAAAAAACAAAATAATCTGGTCGATTTCAGAGAAAGACAATTTCGTTT |
| Zms3 | ATGCCCATTTAAAACATCATGAATCCATGTCTTGATGGCAGGATAAGTGAAAGAAAATGGGTGTTCACAAAATTGCCCTAGCATGACAGAATAAAATTCTTTATATCCTATCGTGGAAACACTGCATGAAAGATCGATTCTGATCAATGTAAGGTTTCCATTTCGTAAAATGGCGTAATTTTGTTAGCGGAAAGATGCTTTCCGTTGACCCTTGCCGTTATCGTCGCTATAGCGCCCGTCTACATCTCCTGTTGACGGTGAATCTTTGGCGTCAGACGGGTGCGCTTGAACATTGCCATATATGCGCGTCTTCCTTTTAAAGAATTCACGCAGGCGAGCTTAGTCATTTTTGCTCGGTGCTATTTTCATAATTTAATTATGGTCAGGCGCATTTTGTATATTTGGTAAAGTAACTCTTGAGGTGAAGGGCTTCATGCCGACGATTAATCAATTGGTCCGTAAGGGCCGTCA |
| Zms4 | CACGAGCTCAGAAGTTTTCTGCATTGTCTCCGAATTCCTTGCCCTGCTTTCTGTGGCAAAGGGATTCAGAAATTAAACCCTACGAAAACCACAATGCCTCCGAAGCCCATTTCGGAGGCATTATGTTTCAGTCTTATTGGGCTGTCAGCTTTTTGGCCAATTCGGCCACATGCTTGCCTAAGAATTTCGCGCCATCCAACTCATTCTTGCTAGGCTGCCGTGAACCATCGCTTGCGGCAATGGTCGTCGCGCCATAGGGCGCACCACCTGTTACTTCAT |
| Zms6 | TTTTTCTTTTTCAATCAACTTTAATGATGAAAAAAAATACAAAAGAAGTTTTTATCAAAAGAGCGGGATAGGGGGATTTTGGTAGCCGTTTTTTTTACAAGACAAGAATGAGAGAGTGGTTCGCAACTAATGTTGCATCTATAACACATTTCTCGCCCATTTTAGCACAGACATGAAAAGCACGATGACAAGATCGCAGAAGTCGCATAGCCTTCAATACAGGCCTTAATTCAAGGGGGATTGGTCTAAAAGACTAAAGTCTTTTTATGATTCTTTCGGGGGGAAGTAATCAAAAGACTATATG |
| Zms8 | AAAATGGAGCAAGAGGAAAAAGAACGACTGGCAGTTTTGATCGAAGACTATGCAGAAAGAATGGAAAATATCGTAAATCGCCTTGATGATGAAGCGCAAGCATCCTAAATAGGCAGACGGCGGTTCTGCGCGGTGCGCGCTCTAGCAATTATCCCTGAGGCTATAAATACATCCAAGGGAGCTGTCCCTGTTCGAATCCTGGTTCGATGCATACGGCGCCCACCTGACGTTTGGGCGTCAGTGGATATTCAAGCAAACGGCCTGGCAGCGGTTCCGCCACTTTAATATACAGGTCAATGACACTGTTTTTCTTAGAATGACCGCATCTAATAGCGGATTATAATTCTGCGGTCTCTCTTATCGCTCTTCAAACCATACGGACAGAGCATTGGTCTAACTAACCAGACTAATCGACCCGAAATATTTCTCAGCATCGTTAACTGTCGAGCTAAATAGCGATGGGGCTTCACAAAAAACAGGTCACCCTTTTTCTATTTTGCATTCTCATACCTACCCGTCTCAAATCGGCTTAGAATTTTCAGCGAACAGACAAAAAAATCCCTGTTCTGACGAACAGAACAGGGACAGGGAACAAGGGTAAACTAGGGAGTAAGCAAAATAAGAATTATTCCGCGGCAGGAAGGTAGATTT |
| Zms9 | GTATTGGATGTTTAATAAGCCGAAGCAGTTCAGGCGCATTGCCACACGCTATGACAAAACCAGAAAATCTTTCCTCGCCTTCCTTCATCTCGTGGCCGTAAAATTGTGGTTGCCTTCCTTTGTCAACAAAACCTAGGATTTCAAGAAGACCATTCCAGACGCTCTGTTCTGTCCTTCTTTTGAACTGAACATAAAGCGAATTCCATTCCCATAGTATTTCATGATGTCTCGTCATGAGCAGCTCGTTCGAGACACATAAATCACCCCGTTAAGAATATAACAATATAGGGATGAGCGCGACCAGCGATTGTATTCGTTTGGAAGAACACGCTCTATTCCTTCCACGTGGTGTCCGCCAGATTCCCACTCATCCAGTGTGTCCTGTAAAAACTTGATATCAAATTATGCTTTCAAAGCTTGAACTATATACTTTATCAACATAACTTAGTCTACAAAAAGACCGTAAAAATAATTTTTTAAGTATTGTAATTAATATCAGAA |
| Zms10 | CTTTCAGGCGGACAAAAAAGCCGGAGACTTTTTTCAGGCTGTCGAGCTTGCCATGTGGTATCGCAATACCATGACCAAAACCTGTCGAACCGAGCGTTTCTCTTTCATGTAACCGTTCGGCGATAACCTTGGGGTTAATTAGAAATTCTCGCCCAGCCCATTGGGCAACCTGCTGGAAGAGTGTTTTTTTGTTAGAAGGATTGAAATTCACCTCAATCCTGTCCGGCGCGATTATATCGGCCAGTTCATTCATGTTTTTTCGCCTTTATATGCCCACGATTGCAAAGATCGCAGCATCAGACATTTTCTGAAAGACACAGCCTTTCAGAAAATAAAGTCAAAATTCTAGCAATATTCCTTCCGGAAACGGGGGAGAGGGGTCGATAATTCTCTTGAAAAGAGGGAGCGACCTCTCTCCCGTTTATTATCTCCGTCTATCTGAATATTTAACGACTATCGCGGCTCAACCCAACCGATGGTACCATCTACCCGACGATAAACCATATTATGATGACCCGATTTGCTGTTCACGAAGAGCAAAGCCGTCGTGTTGCGAAGATCGAGCATCAAAACAGCATCGGAAACGCTGGCTGTTGGAATATCAACCTGCATTTCGGCAATGACGGGAGGATGATCGCCTT |
| Zms13 | AAAGCCAGTTCAGTTTTGATTGATAAGCTAACAGATTGATATTCATCAGTAAGACGGCAGAAGCGCTAAAAGAGACTTTTGCTGAAAATCAGTGTGGGCAAATACATAGATAGAAAAAGCCATGATGGCTTTTGATAGTAAAAATGGGAAGCTTCTGTTGCCCGGTGCTTCCCGAACCGCTGGAATCTGCTTCTGTTGCCCGGTGCAGCCCAACCGCGCTCTTACTTAGTAATGTCTACCCGTGAAGGTTTCCATTACGCAGCAACAGCGAGTACCTCGTTATCGTTGGCACTTATAAAAATGATCGGTTTAACGGCTCAATCACGCCGGATGGCAATCAAGTCTTTACTACACACGTCGATCCTGTTTCGCCCCCATCAACAACAGTGGCTTGCTATCCGCGGCTGTTGGTGGAGGCGCCGGAGTACTGCCCTCCGGGTCCGTCGAGTCTATTTCACTTAACAGTCTACCATCGTAGTCACCGAAGTGACGCTCTCTAGCTAAGGTCGGACAGCCATAAAGGCAATAGCTGTCCGTAAATTTTTATAAAAACTTTGCATGATTACTTGCAGCAGTCGCTTCTTCTTTGACTTTCTCCGCTGGATTCGGCTCTTTTTCTGATGGAT |
| Zms15 | GATATGGCTGCCGTGTGTCGGTGGCCTTTTTTAACAATCAAGAAATACTGTTGAGGAATGAGCGATGAAGCGCACTTTTCAGCCGAGCAACCTCGTGC |
| Zms16 | ATAATTTTCCGTTGTTACGGGCTTGCAAACCCCGTCTGAATGTTTCATAGGCGATAGTGGAAGTCGGGCGGACGGTGCTGTCGCCAACCCGGTCAGGTCCGGAAGGAAGCAGCCGTAACGAATTTGTATCGGGTCGTTCCGGCTTCCACGTCTTAAAGATTTCTAAAAATTCCTTTTTTCTTTATCCTTGATAATCGCGCTTTTATTCCGTTTCGGATAGATGTCCTTTTAAAGCCGGATAAGAATGATGGGCTGAAAAATATTTCAGTCGCTTTCCTGCCCCATATTATGCATTAACTTCGTCCTATGGTTGATTCTTCCTCTCT |
| Zms18 | GATTTTTGAATGACTGCTTCGGTGGTCGTTTAGAAAGCGAAAGTTGCAAAAGTAAAGATATTCAATTGATGCTGAGATTTAACAAAAAGCATCATCGGTGGAAGAAGCAATTCTGACATTGATGGTGGAAGTTCTCAAGCGTGAGGTAAGAGCATTTGGTGGATGCCTAGGCATACACAGGCGATGAAGGACGTGGCACGCTGCGATAAGCTACGGCGAGATGTGAGCAATCTTTGACCC |
| Zms20 | GTCATACCAGCAAGGCCGCTTATTGGAGATAGTTAAGCTGCTATAAATATTATATTTTACAAAATAATGCTTTCTGATTGATTTCGGGGAAAAAGGTTATTCCGACCTTTCTTTTCTTTTGCAGTGTTCTGTCAGCCTGAAAATATCTATAATAGCTGTTTTCAGGTTGCCTTTTCCTAACGCATGCAACCGAAAACGAGGCTTGGGGTTTAGCCTTGCAAGAATAAAAAGAAGGAAGCGAGAAAGAGAACGGTATGCGCGTTTTAATCGTTGAGGATGAGCCGACGCTTTCCCGTCAGTTGCGAACAACTTTGGAAGGG |
| negsRNA302 | ATTGCCAATGGGCCGGTAGCTCAGGTGGTTAGAGCGCACGCCTGATAAGCGTGAGGTCGGAGGTTCAACTCCTCCCCGGCCCACCATGGTTCAGGTAGGGAATTTCAAAGGGGCCTTAGCTCAGTTGGGAGAGCGCTAGCTTTGCAAGCTTGAGGTCATCGGTTCGATCCCGATAGGCTCCACCAGAAATTATCTGGTTGT |
| negsRNA355 | ATGTTTTCTCTTAGCTCTTTTGAATATCTTCGATTTTCAATTAACTTCACGCACAGGTGTCATAGCTTGGTGGCTATAGCGTCAGTGACCCACCCGATCCCATTTCGAACTCGGACGTGAAACCTGTCCTGCGCCGATGGTACTGTTGCTTAAGCAACGGAAGAGTAGGTCGTCGCCAGGCTTTGTCACTTGTGCTTGAATTTCCA |
| possRNA305 | AGTTGCAAGGGCAAAGGTCATCGGTCTGCAAAGCATCCAGAACGCGCAAGGCTTCTGCCGGATTACGGCCAACATTCAAAGAGTTGACCGTCACATGCTGAATGACATTGTTCGGATCAACAATGAAGGTTGCGCGATAGGCAACGCCTGCTTCTTCATTCACAATACCCAAAGCAGAGGCCAGCTTTTTGGAATTATCTGCAATCCACGGGAAGTCAGCATTTTCCAAACGTTCGTCAGATTTCCGCCAAGCGTAATGGACGAAAGCGGTATCACAAGAACCACCGATCAGAACGGCATCGCGCAAAGACAGGTCTTCTTTGATATCGTTATAGCCCACAATTTCGGTCGGGCAGACAAAAGTGAAATCCATCGGCCAAAAAAAGACGACCTTCCACTTGCCATTTGTT |
| negsRNA78 | GGACAAAGCCGGTTTCAAAAGGTTAGAGGCATCCATTGCCCCGCCCGAGGTTGCGCCAGCGCCTATAATCGTATGGATTTCATCGATAAACAAAACAGCTTTCGGCTGTTTTTGCAGTTCGGTTACGACGGCTTTCAGTCTTTCTTCAAAATCCCCACGATAACGGGTGCCAGCCAGCAATGCCCCCATATCCAAAGAATA |
| possRNA223 | CTTATAGTTTTATTTTCTCTTTAAAAATTTATCTTATTTCCGATCAATCGGTTATATTTCGCCTGATAGATTCAAGACATTCTGTATTGGGATTGTCTGAATATATTTCGGGTAGATCTGTTTAAAGTGGTTTTTTTGTCCACTTTCCTAATTAGGTTATTTCCATAACGTGACCCAATAGCGTGGCCGTGAGCGCTTGGC |
| negsRNA212 | CTGGACAAGACCCGAATACATTCTTATACGCCCACCCAGACGAGAGAAAAACTCTCAAGCCCAGGTAGCTCAGTCGGTAGAGCATGTGACTGAAAATCACAGTGTCGGCGGTTCGATTCCGTCCCTGGGCACCACTCACATTCCCTAAAAATTTATCTGAAATACAAACTGCCCCTCATGCGCAGATTATTTTTCTATTTC |

## REFINE Pipeline User Guide

Starting Point: Transcriptome .bam files for two different conditions (condition1.bam and condition2.bam)

Additional Necessary Files: A reference fasta file for genome (genome.fa) and your initial .gff file (genome.gff)

Necessary Programs: samtools, bedtools

**
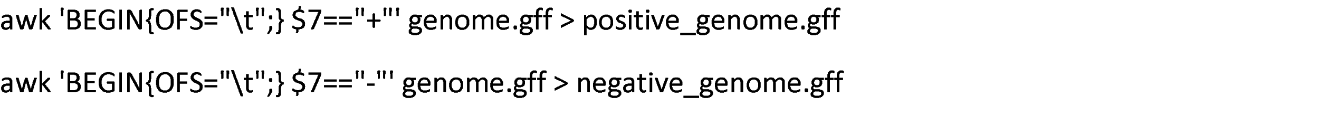
**

### Step 1: Create stranded intergenic .gff files

- Generate an index .fai file from your reference fasta file using the following command:

**
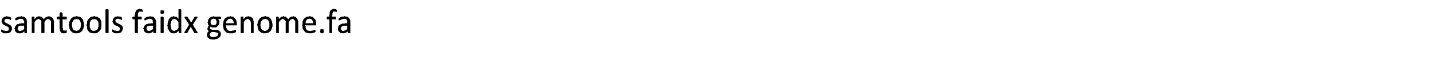
**

- Take your new file (genome.fa.fai) and use it to create a genome .txt file

**
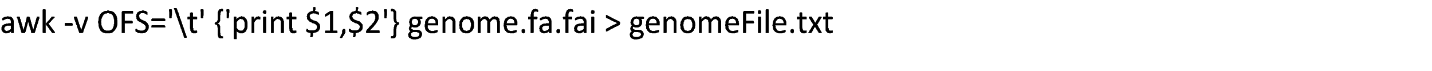
**

- Separate your .gff file by direction into two separate files

**
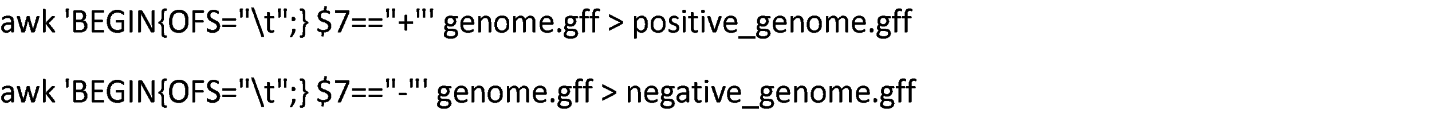
**

- Use bedtools complement to find the intergenic regions

**
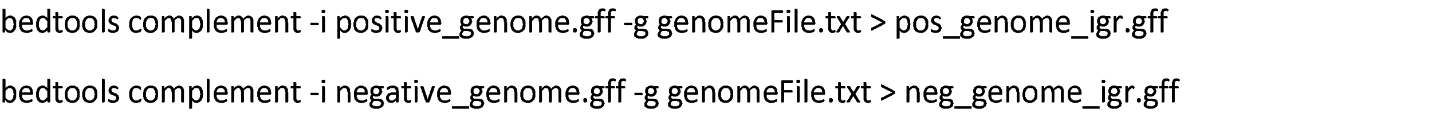
**

### Step 2: Convert your .bam files to .txt tab-delimited file format

- Take your two .bam files and run the following:

**
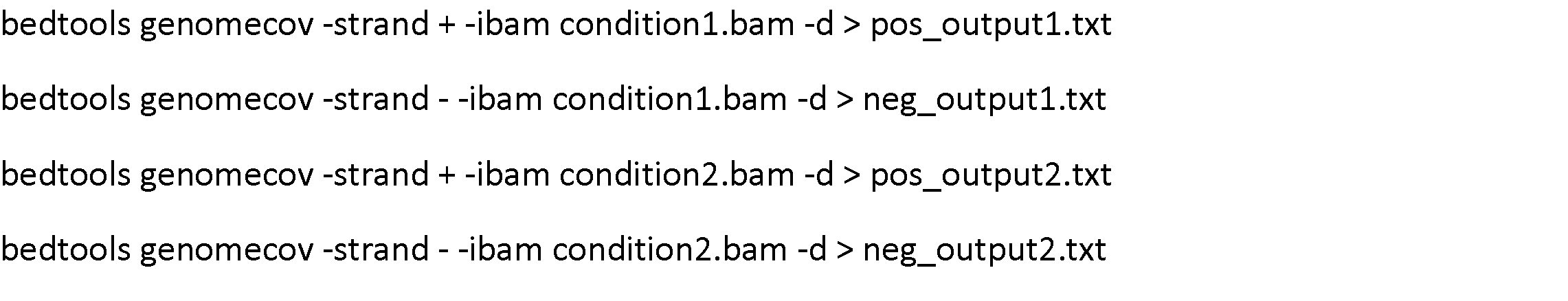
**

### Step 3: Use sRNAScout to locate predicted regions

- Open files.R. Edit the file so that each line contains the ABSOLUTE path to all necessary files.
- Pick appropriate variables for your dataset. You can pick your own variables or run suggest_vars() to get recommended values.
  - min_exp = Minimum level of expression required in a region
  - length_min_exp = Number of nucleotides in a row that a region must be above the minimum expression level.
  - min_de = Minimum differential expression level between reads
  - length_de = Number of reads in a row that must be above the minimum differential expression
  - OPTIONAL: reads_length1 = TOTAL number of reads in your initial condition 1 file. Used for normalization
  - OPTIONAL: reads_length2 = TOTAL number of reads in your initial condition 1 file. Used for normalization.
  - OPTIONAL: distance = how near two regions need to be together to be combined into one continuous region.
- Create the intaRNA input query file using the finalize() function with the variables you have chosen as well as the following file paths:
  - fastaFile = /path/to/genome.fa
  - OPTIONAL: /path/for/output/file. Default is ~/intaRNAInputs.fasta.
- Create the intaRNA target file using the intarna_fasta() function with the following inputs:
  - fastaFile = /path/to/genome.fa
  - gene_names = /path/to/gene_names.txt
  - gene_locs = /path/to/gene_start_locs.txt
  - OPTIONAL: /path/for/output/file

### Step 4: Run IntaRNA

- If you are running a large number of regions, generate the accessibility data:

**
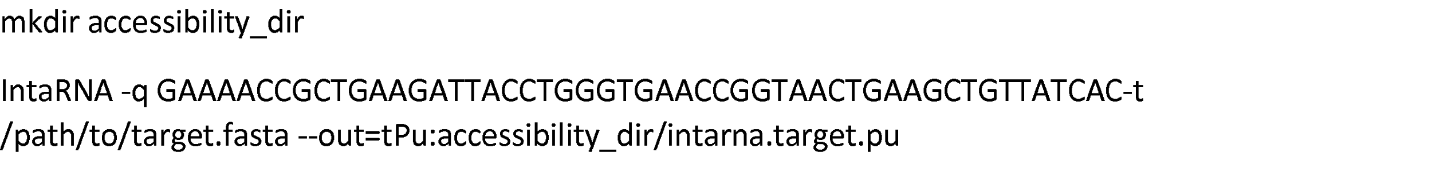
**

- Run IntaRNA using the following command:


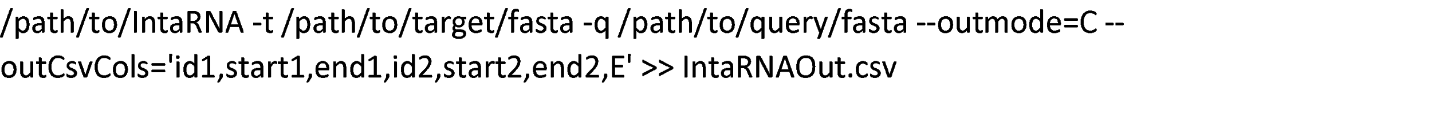


### Step 5: Calculate the sRNAScores and transScores

- Create a custom gff using the finalize() function in func2_dplyr.R
- Generate htcount count files using two name-sorted sam files and your custom gff:


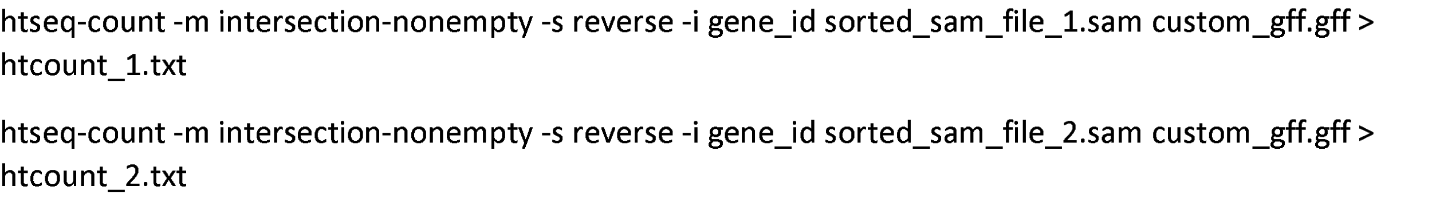


- Run the 2016-08-31 DESeq2_aero_anaero.R script with the two new htcount files to generate an Excel sheet with information for the transScores.
- Run IntaCalc.R using the output from the 2016-08-31 DESeq2_aero_anaero.R and the output from intaRNA.
- Sort the outputs from IntaCalc to get the top 50 sRNAScores or transScores.

#### files.R

# The location of this folder.

workingDir = "D:/REFINE"

setwd(workingDir)

#The name of your genome to be used for descriptive file names.

template="k12"

fastaFile="ZM4.fa"

gffFile="D:/k12_srna.gff"

# Positive output from bedtools genomecov for condition 1

pos_out_1="D:/sRNApredict_MEG/Intergenic_Regions/pos_k12_output1.txt"

# Positive output from bedtools genomecov for condition 2

pos_out_2="D:/sRNApredict_MEG/Intergenic_Regions/pos_k12_output2.txt"

# Negative output from bedtools genomecov for condition 1

neg_out_1="D:/sRNApredict_MEG/Intergenic_Regions/neg_k12_output1.txt"

# Negative output from bedtools genomecov for condition 2

neg_out_2="D:/sRNApredict_MEG/Intergenic_Regions/neg_k12_output2.txt"

# Positive intergenic gff from bedtools complement

pos_inter_gff="D:/sRNApredict_MEG/Intergenic_Regions/k12_pos_intergenic.gff"

# Negative intergenic gff from bedtools complement

neg_inter_gff="D:/sRNApredict_MEG/Intergenic_Regions/k12_neg_intergenic.gff"

source("func2_dplyr.R")

source("toolBox.R")

#### func2_dplyr.R

library("dplyr")

library(magrittr)

library(GenomicRanges)

source("https://bioconductor.org/biocLite.R")

biocLite("Biostrings")

require(Biostrings)

source("D:/sRNApredict_MEG/Intergenic_Regions/OutputToInter.R")

source("D:/sRNApredict_MEG/sRNA_Predict2/toolBox.R")

pos_inter <- intergen(pos_out_1, pos_out_2, pos_inter_gff)

neg_inter <- intergen(neg_out_1, neg_out_2, neg_inter_gff)

#pos_inter1 <- read.csv("D:/sRNApredict_MEG/sRNA_Predict2/pos_k12_inter1.csv", header=TRUE, sep = ",", na.strings=c("","NA"), stringsAsFactors=FALSE, colClasses = c("character", "character", "integer", "integer"))

#pos_inter2 <- read.csv("D:/sRNApredict_MEG/sRNA_Predict2/pos_k12_inter2.csv", header=TRUE, sep = ",", na.strings=c("","NA"), stringsAsFactors=FALSE, colClasses = c("character", "character", "integer", "integer"))

#neg_inter1 <- read.csv("D:/sRNApredict_MEG/sRNA_Predict2/neg_k12_inter1.csv", header=TRUE, sep = ",", na.strings=c("","NA"), stringsAsFactors=FALSE, colClasses = c("character", "character", "integer", "integer"))

#neg_inter2 <- read.csv("D:/sRNApredict_MEG/sRNA_Predict2/neg_k12_inter2.csv", header=TRUE, sep = ",", na.strings=c("","NA"), stringsAsFactors=FALSE, colClasses = c("character", "character", "integer", "integer"))

directions <- c("+", "-")

#function with arguments

# a - number for designated high expression

# b - length of high expression area to qualify region

# c - number fold to look for between aerobic and anaerobic differential expression

# d - length of differential expression to qualify region

# returns a list of the row numbers

higherThan <- function(min_exp, length_min_exp, min_de, length_de, direction, reads_length1=NA, reads_length2=NA, distance=50, utr_region_length=NA, max_length=500){

if (max_length < length_min_exp){

warning("The entered value for length_min_exp is greater than the set maximum of ", max_length, ". Maximum changed to ", 2*length_min_exp, ".")

max_length=length_min_exp*2

}

# clean data

if (direction == "+") {

inter1 <- filter(pos_inter$inter1, !grepl(".", V2, fixed = TRUE)) # removes duplicate rows generated by OutputToInter.R. These are labeled as nt 1.1, 2.1, 3.1, etc

inter2 <- filter(pos_inter$inter2, !grepl(".", V2, fixed = TRUE))

inter1 <- inter1[which(!is.na(inter1$V2)),]

inter2 <- inter2[which(!is.na(inter2$V2)),]

} else {

inter1 <- filter(neg_inter$inter1, !grepl(".", V2, fixed = TRUE)) # removes duplicate rows generated by OutputToInter.R. These are labeled as nt 1.1, 2.1, 3.1, etc

inter2 <- filter(neg_inter$inter2, !grepl(".", V2, fixed = TRUE))

inter1 <- inter1[which(!is.na(inter1$V2)),]

inter2 <- inter2[which(!is.na(inter2$V2)),]

}

if (!is.na(reads_length1) && !is.na(reads_length2)){

norm_ratio = reads_length1/reads_length2

inter2$V3 <- inter2$V3 * norm_ratio

} else {

norm_ratio = 1

}

inter <- left_join(inter1, inter2, by = c("V2", "V1", "V2")) %>%

na.omit() %>%

dplyr::rename(nt = V2) %>% # nucleotide number

dplyr::rename(chr = V1) %>% # chromosome name

dplyr::rename(Exp1 = V3.x) %>% # Expression level, sample 1

dplyr::rename(Exp2 = V3.y) %>% # Expression level, sample 1

select(nt, chr, Exp1, Exp2) # get rid of duplicate columns by selecting the renamed ones

inter$nt <- as.numeric(inter$nt) # convert nt from chr to numeric to allow sorting

# differential expression filters

#differential fold minimum

de1 <- filter(inter, min_de*inter$Exp1 <= inter$Exp2 & inter$Exp2 != 0)

de2 <- filter(inter, inter$Exp1/min_de >= inter$Exp2 & inter$Exp1 != 0)

de <- bind_rows(de1, de2) %>%

arrange(nt)

# minimum expression level filter

min_exp_filt <- filter(inter, inter$Exp1 >= min_exp | inter$Exp2 >= min_exp)

# first nt of each sequential region

region_starts_de <- c(1, which(diff(de$nt, lag = 1) != 1), length(de$nt))

inter_starts <- inter$nt[c(1, which(diff(inter$nt, lag=1)!=1), length(inter$nt))+1]

# find regions that may contain UTR's

utr_regions <- c()

if (!is.na(utr_region_length)){

inter_ends <- inter$nt[c(which(diff(inter$nt, lag=1)!=1), length(inter$nt))]

for (i in 1:length(inter_starts)){

if(is.na(inter_starts[[i]])){

print("True")

break

}

utr_regions <- c(utr_regions,

seq(inter_starts[[i]], inter_starts[[i]]+utr_region_length),

seq(inter_ends[[i]]-utr_region_length, inter_ends[[i]]))

}

}

# sequential diff exp nt in list of lists

de_regions <- sapply(seq(length(region_starts_de) - 1), function(i) de$nt[(region_starts_de[i] + 1):region_starts_de[i+1]])

# gather only regions >= length_de

de_regions_of_length = list()

for (x in de_regions){

if(length(x) >= length_de){

de_regions_of_length = c(de_regions_of_length, list(x))

}

}

# find sequential regions with min exp for at least length_min_exp

region_starts_min <- c(1, which(diff(min_exp_filt$nt, lag = 1) != 1), length(min_exp_filt$nt))

# sequential min exp nt in list of lists

min_exp_regions <- sapply(seq(length(region_starts_min) - 1), function(i) min_exp_filt$nt[(region_starts_min[i] + 1):region_starts_min[i+1]])

# gather only regions >= length_min_exp and splits up large regions at their lowest trough

min_exp_regions_of_length = list()

for (x in min_exp_regions){

last_min_loc = 1

if(length(x) >= length_min_exp && length(x) < max_length){

min_exp_regions_of_length = c(min_exp_regions_of_length, list(x))

} else if (length(x) > max_length) {

last_min = x[1]

chunk_size = as.integer((2*length(x)/max_length))

# finds lowest point in each split up region

for (step in 0:chunk_size){

start_loc = step*max_length/2 + 1

end_loc = min(((step+1)*max_length/2 + 1), length(x))

nt_locs = which(inter$nt %in% x[start_loc:end_loc])

temp_nt = inter$nt[nt_locs]

min_loc = which.min(inter$Exp1[nt_locs])

if (temp_nt[min_loc] > last_min) {

min_exp_regions_of_length = c(min_exp_regions_of_length, list(seq(last_min, temp_nt[min_loc])))

last_min = temp_nt[min_loc] + 1

}

}

}

}

# make GRanges objects

de_granges <- data.frame(chr = character(length(de_regions_of_length)),

start = integer(length(de_regions_of_length)),

end = integer(length(de_regions_of_length)),

strand = character(length(de_regions_of_length)),

stringsAsFactors = FALSE)

for (i in 1:length(de_regions_of_length)) {

de_granges$chr[i] <- "chromosome"

de_granges$strand[i] <- "*"

de_granges$start[i] <- head(de_regions_of_length[[i]], 1)

de_granges$end[i] <- tail(de_regions_of_length[[i]], 1)

}

min_exp_granges <- data.frame(chr = character(length(min_exp_regions_of_length)),

start = integer(length(min_exp_regions_of_length)),

end = integer(length(min_exp_regions_of_length)),

strand = character(length(min_exp_regions_of_length)),

stringsAsFactors = FALSE)

for (i in 1:length(min_exp_regions_of_length)) {

min_exp_granges$chr[i] <- "chromosome"

min_exp_granges$strand[i] <- "*"

min_exp_granges$start[i] <- head(min_exp_regions_of_length[[i]], 1)

min_exp_granges$end[i] <- tail(min_exp_regions_of_length[[i]], 1)

}

de_GR <- makeGRangesFromDataFrame(de_granges)

min_exp_GR <- makeGRangesFromDataFrame(min_exp_granges)

distn = distanceToNearest(min_exp_GR, de_GR)

hits <- as.data.frame(distn)

hits_filt <- hits %>%

filter(distance <= 0)

sRNA_pred_regions <- data.frame(chr = character(nrow(hits_filt) + 1),

start = integer(nrow(hits_filt) + 1),

end = integer(nrow(hits_filt) + 1),

mid = integer(nrow(hits_filt) + 1),

strand = character(nrow(hits_filt) + 1),

stringsAsFactors = FALSE)

final_sRNA_pred_regions <- list()

i_count=1

for (i in 1:length(hits_filt$queryHits)) {

region_start = min(min_exp_granges$start[hits_filt$queryHits[i]], de_granges$start[hits_filt$subjectHits[i]])

region_end = min(min_exp_granges$end[hits_filt$queryHits[i]], de_granges$end[hits_filt$subjectHits[i]])

if (!region_start%in%utr_regions && !region_end%in%utr_regions){

final_sRNA_pred_regions$chr[i_count] <- "chromosome"

final_sRNA_pred_regions$strand[i_count] <- "*"

final_sRNA_pred_regions$start[i_count] <- region_start

final_sRNA_pred_regions$end[i_count] <- region_end

i_count=i_count+1

}

}

final_sRNA_pred_regions$start[final_sRNA_pred_regions$start < 0] <- 0

bed_sRNA <- vector()

# Combines nearby regions with reasonably similar expression levels

i = 1

while (i <= length(final_sRNA_pred_regions$start)){

final_end = final_sRNA_pred_regions$end[length(final_sRNA_pred_regions$end)]

i_count = 0

curr_start_loc = which(inter1$V2 %in% final_sRNA_pred_regions$start[i])

curr_end_loc = which(inter1$V2 %in% final_sRNA_pred_regions$end[i])

new_max = max(inter1$V3[curr_start_loc:curr_end_loc])

new_min = new_max

while (i+i_count < length(final_sRNA_pred_regions$start) && (final_sRNA_pred_regions$start[i+i_count+1] - final_sRNA_pred_regions$end[i+i_count]) < distance){

inter_end_loc = which(inter1$V2 %in% final_sRNA_pred_regions$start[i+i_count+1])

peak_end_loc = which(inter1$V2 %in% final_sRNA_pred_regions$end[i+i_count+1])

new_max = max(new_max, max(inter1$V3[inter_end_loc:peak_end_loc]))

new_min = min(new_min, max(inter1$V3[inter_end_loc:peak_end_loc]))

if (new_min/new_max <= 0.02){

break

}

i_count = i_count + 1

}

bed_sRNA <- c(bed_sRNA, seq.int(final_sRNA_pred_regions$start[i], final_sRNA_pred_regions$end[i+i_count]))

i = i + i_count + 1

}

outputlist = which(inter1$V2 %in% bed_sRNA)

output <- list("outputlist"=outputlist, "inter1_var"=inter1, "inter2_var"=inter2)

temp_output <- inter1$V2[outputlist]

return(output)

}

#function that takes argument of row numbers, alters inter1 and returns a new table with just those rows

tableOne <- function(input_list){

inter1_var = input_list$inter1

x = input_list$outputlist

newinter1 = inter1_var[x, ]

newinter1 = newinter1[,3:4]

colnames(newinter1) = c("loc", "expr")

return (newinter1)

}

#function that takes argument of row numbers, alters inter2, and returns a new table with just those rows

tableTwo <- function(input_list){

inter2_var = input_list$inter2_var

x = input_list$outputlist

newinter2 = inter2_var[x,]

newinter2 = newinter2[,3:4]

colnames(newinter2) = c("loc", "expr")

return (newinter2)

}

#takes in a data frame with "loc" heading

#returns a list of lists containing the sequential regions

#used in num() min() and max() functions in Server.R

getRange <- function(table){

interlist1 = table$loc

interBreaks1a <- c(0, which(diff(interlist1, lag = 1) != 1), length(interlist1))

interBreaks1b = sapply(seq(length(interBreaks1a) - 1), function(y) interlist1[(interBreaks1a[y] + 1):interBreaks1a[y+1]])

return(interBreaks1a)

}

#### toolbox.R

htcount <- function(min_exp, length_min_exp, min_de, length_de, outFile){

pos_higher = higherThan(min_exp, length_min_exp, min_de, length_de, "+")

neg_higher = higherThan(min_exp, length_min_exp, min_de, length_de, "-")

pos_output = getRange(tableOne(pos_higher))

neg_output = getRange(tableOne(neg_higher))

for (i in 1:length(neg_output)){

output_locs = which(neg_higher$inter1_var$V2 %in% neg_output[[i]])

print(sum(neg_higher$inter1_var$V3[output_locs]))

center = as.integer(length(neg_output[[i]])/2)

dnastr=""

if (length(neg_output[[i]]) > 200){

for (j in 1:length(neg_output[[i]])){

dnastr = paste(dnastr, sequence[[1]][neg_output[[i]][j]],sep="")

}

i_count = i_count + 1

} else {

if (length(neg_output[[i]]) > 6){

for (j in -100:100){

dnastr = paste(dnastr, sequence[[1]][neg_output[[i]][center]+j],sep="")

}

i_count = i_count + 1

}

}

}

i_count = 1

for (i in 1:length(pos_output)){

dnastr=""

center = as.integer(length(pos_output[[i]])/2)

if (length(pos_output[[i]]) > 200){

for (j in 1:length(pos_output[[i]])){

dnastr = paste(dnastr, sequence[[1]][pos_output[[i]][j]],sep="")

}

i_count = i_count + 1

} else {

for (j in -100:100){

dnastr = paste(dnastr, sequence[[1]][pos_output[[i]][center]+j],sep="")

}

i_count = i_count+1

}

if (any(pos_output[[i]] %in% flattened_pos_list)){

lapply()

}

lapply(dnastr, write, outFile)

}

}

# TODO: add a descriptor variable to add a description to the file name

finalize <- function(min_exp, length_min_exp, min_de, length_de, fastaFile) {

neg_output_full <- list()

myFastaFile = readDNAStringSet(fastaFile)

sequence = paste(myFastaFile)

sequence = strsplit(sequence, NULL)

pos_output = getRange(tableOne(higherThan(min_exp, length_min_exp, min_de, length_de, "+")))

neg_output = getRange(tableOne(higherThan(min_exp, length_min_exp, min_de, length_de, "-")))

i_count = 1

for (i in 1:length(neg_output)){

if (any(neg_output[[i]] %in% flattened_neg_list)){

print(i_count)

}

center = as.integer(length(neg_output[[i]])/2)

dnastr=""

if (length(neg_output[[i]]) > 200){

for (j in 1:length(neg_output[[i]])){

dnastr = paste(dnastr, sequence[[1]][neg_output[[i]][j]],sep="")

}

# lapply(paste(">NegSeq", i_count, sep=""), write, "D:/EthanolIntaRNAInputs/intaRNANegRef.fasta", append=TRUE)

# lapply(seq(max(0,neg_output[[i]][[center]]-100),neg_output[[i]][[center]]+100, sep="\n"), write, "D:/EthanolIntaRNAInputs/intaRNANegRef.fasta", append=TRUE)

# lapply(dnastr, write, "D:/EthanolIntaRNAInputs/intaRNANegRef.fasta", append=TRUE)

# lapply(paste(">NegSeq", i_count, sep=""), write, "D:/EthanolIntaRNAInputs/intaRNANeg.fasta", append=TRUE)

# lapply(dnastr, write, "D:/EthanolIntaRNAInputs/intaRNANeg.fasta", append=TRUE)

i_count = i_count + 1

} else {

if (length(neg_output[[i]]) > 6){

for (j in -100:100){

dnastr = paste(dnastr, sequence[[1]][neg_output[[i]][center]+j],sep="")

}

# lapply(paste(">NegSeq", i_count, sep=""), write, "D:/EthanolIntaRNAInputs/intaRNANegRef.fasta", append=TRUE)

# lapply(seq(max(0,neg_output[[i]][[center]]-100),neg_output[[i]][[center]]+100, sep="\n"), write, "D:/EthanolIntaRNAInputs/intaRNANegRef.fasta", append=TRUE)

# lapply(dnastr, write, "D:/EthanolIntaRNAInputs/intaRNANegRef.fasta", append=TRUE)

# lapply(paste(">NegSeq", i_count, sep=""), write, "D:/EthanolIntaRNAInputs/intaRNANeg.fasta", append=TRUE)

# lapply(dnastr, write, "D:/EthanolIntaRNAInputs/intaRNANeg.fasta", append=TRUE)

i_count = i_count + 1

}

#}

}

}

i_count = 1

for (i in 1:length(pos_output)){

dnastr=""

center = as.integer(length(pos_output[[i]])/2)

if (length(pos_output[[i]]) > 200){

for (j in 1:length(pos_output[[i]])){

dnastr = paste(dnastr, sequence[[1]][pos_output[[i]][j]],sep="")

}

lapply(paste(">PosSeq", i_count, sep=""), write, "D:/EthanolIntaRNAInputs/intaRNAPos.fasta", append=TRUE)

lapply(dnastr, write, "D:/EthanolIntaRNAInputs/intaRNAPos.fasta", append=TRUE)

lapply(paste(">PosSeq", i_count, sep=""), write, "D:/EthanolIntaRNAInputs/intaRNAPosRef.fasta", append=TRUE)

lapply(seq(max(0,pos_output[[i]][[center]]-100),pos_output[[i]][[center]]+100, sep="\n"), write, "D:/EthanolIntaRNAInputs/intaRNAPosRef.fasta", append=TRUE)

lapply(dnastr, write, "D:/EthanolIntaRNAInputs/intaRNAPosRef.fasta", append=TRUE)

i_count = i_count + 1

} else {

for (j in -100:100){

dnastr = paste(dnastr, sequence[[1]][pos_output[[i]][center]+j],sep="")

}

lapply(paste(">PosSeq", i_count, sep=""), write, "D:/EthanolIntaRNAInputs/intaRNAPos.fasta", append=TRUE)

lapply(dnastr, write, "D:/EthanolIntaRNAInputs/intaRNAPos.fasta", append=TRUE)

lapply(paste(">PosSeq", i_count, sep=""), write, "D:/EthanolIntaRNAInputs/intaRNAPosRef.fasta", append=TRUE)

lapply(seq(max(0,pos_output[[i]][[center]]-100),pos_output[[i]][[center]]+100), write, "D:/EthanolIntaRNAInputs/intaRNAPosRef.fasta", append=TRUE)

lapply(dnastr, write, "D:/EthanolIntaRNAInputs/intaRNAPosRef.fasta", append=TRUE)

i_count = i_count + 1

}

#}

}

}

finalize2 <- function(min_exp, length_min_exp, min_de, length_de, fastaFile, outFile, min_size=200) {

neg_output_full <- list()

myFastaFile = readDNAStringSet(fastaFile)

sequence = paste(myFastaFile)

sequence = strsplit(sequence, NULL)

pos_output = getRange(tableOne(higherThan(min_exp, length_min_exp, min_de, length_de, "+")))

neg_output = getRange(tableOne(higherThan(min_exp, length_min_exp, min_de, length_de, "-")))

i_count = 1

for (i in 1:length(neg_output)){

center = as.integer(length(neg_output[[i]])/2)

dnastr=""

if (length(neg_output[[i]]) > min_size){

lapply(paste("Chromosome", "ena", "sRNA", neg_output[[i]][[1]], neg_output[[i]][[length(neg_output[[i]])]],".","-",".",

paste("ID=gene:negsRNA", i, ";description=small_rna;gene_id=negsRNA", i, sep=""), sep="\t"),

write, outFile, append=TRUE)

for (j in 1:length(neg_output[[i]])){

dnastr = paste(dnastr, sequence[[1]][neg_output[[i]][j]],sep="")

}

i_count = i_count + 1

} else {

# cuts off regions smaller than 6 nucleotides long because intaRNA does not accept regions this short.

if (length(neg_output[[i]]) > 6){

lapply(paste("Chromosome", "ena", "sRNA", max(0,neg_output[[i]][[center]]-100),neg_output[[i]][[center]]+100,".","-",".",

paste("ID=gene:negsRNA", i, ";description=small_rna;gene_id=negsRNA", i, sep=""), sep="\t"),

write, outFile, append=TRUE)

for (j in -100:100){

dnastr = paste(dnastr, sequence[[1]][neg_output[[i]][center]+j],sep="")

}

i_count = i_count + 1

}

}

}

i_count = 1

for (i in 1:length(pos_output)){

dnastr=""

center = as.integer(length(pos_output[[i]])/2)

if (length(pos_output[[i]]) > min_size){

lapply(paste("Chromosome", "ena", "sRNA", pos_output[[i]][[1]], pos_output[[i]][[length(pos_output[[i]])]],".","+",".",

paste("ID=gene:possRNA", i, ";description=small_rna;gene_id=possRNA", i, sep=""), sep="\t"),

write, "D:/k12_srna.gff", append=TRUE)

for (j in 1:length(pos_output[[i]])){

dnastr = paste(dnastr, sequence[[1]][pos_output[[i]][j]],sep="")

}

i_count = i_count + 1

} else {

lapply(paste("Chromosome", "ena", "sRNA", max(0,pos_output[[i]][[center]]-100),pos_output[[i]][[center]]+100,".","+",".",

paste("ID=gene:possRNA", i, ";biotype=protein_coding;description=small_rna;gene_id=possRNA", i, sep=""), sep="\t"),

write, "D:/k12_srna.gff", append=TRUE)

for (j in -100:100){

dnastr = paste(dnastr, sequence[[1]][pos_output[[i]][center]+j],sep="")

}

i_count = i_count + 1

}

}

}

get_gene_locs <- function(gffFile=gffFile){

gff = read.csv(gffFile, sep="\t", header=FALSE)

gff = filter(gff, grepl("gene", V3, fixed=TRUE))

gff = gff[!duplicated(gff$V4),]

gene_start_locs = gff$V4

gene_names = as.character(gff$V9)

gene_names = unlist(strsplit(gene_names, ";"))

gene_names = gene_names[str_detect(gene_names, "ID=gene:")]

gene_names = gsub("ID=gene:","", gene_names)

output = list(start=gene_start_locs, name=gene_names)

}

intarna_fasta <- function(fastaFile=fastaFile, gffFile=gffFile, template=template) {

myFastaFile = readDNAStringSet(fastaFile)

sequence = paste(myFastaFile)

sequence = strsplit(sequence, NULL)

gene_data = get_gene_locs(gffFile)

start_locs = gene_data$start

gene_names = gene_data$name

start_locs = start_locs$V1

end_locs = start_locs + 100

start_locs = start_locs - 200

start_locs[start_locs < 0] <- 0

end_locs[end_locs > length(myFastaFile[[1]])] <- length(myFastaFile[[1]])

last_start_loc=-1

for (i in 1:length(start_locs)){

if (start_locs[[i]]==last_start_loc){

next

}

dnastr=""

full_loc = seq(start_locs[[i]], end_locs[[i]])

for (j in 1:length(full_loc)){

dnastr = paste(dnastr, sequence[[1]][full_loc[[j]]],sep="")

}

lapply(paste(">",gene_names$V1[i],sep=""), write, paste(template, "fasta.fasta", sep=""), append=TRUE)

lapply(dnastr, write, paste(template, "fasta.fasta", sep=""), append=TRUE)

last_start_loc=start_locs[[i]]

}

}

#### run_DESeq2.R

library("DESeq2")

countdata <- read.csv("C:/Users/kh23462/Dropbox/Contreras Group/REFINE/counts_aero_anaero.csv", header = TRUE, row.names=1)

id <- colnames(countdata)

strain <- c("aero", "anaero")

coldata <- data.frame(row.names=id,strain) #table of sample descriptions (w ID and factors)

coldata$strain = factor(x = coldata$strain, levels = c('aero', 'anaero'))

coldata$strain <- relevel(coldata$strain, "aero")

coldata

ddsMat <- DESeqDataSetFromMatrix(countData = countdata, colData = coldata, design = ~ strain)

#Run DESeq

ddsTC <- DESeq(ddsMat, test="Wald")

resTC <- results(ddsTC)

resTC$symbol <- mcols(ddsTC)$symbol

head(resTC[order(resTC$padj),],4)

data <- plotCounts(ddsTC, which.min(resTC$padj),

intgroup=c("strain"), returnData=TRUE)

#Exporting results

resOrdered <- resTC[order(resTC$padj),]

head(resOrdered)

resOrderedDF <- as.data.frame(resOrdered)

library("xlsx")

write.xlsx2(resOrderedDF, file="2016-08-31 DESeq2_aero_anaero.xlsx")

#exporting specific log2fold changes

res2 <- results(ddsTC, contrast=c("strain","aero","anaero"))

write.xlsx2(res2, file="2016-08-31 DESeq2_aero_anaero.xlsx", sheetName = "aero_anaero_wald", append = TRUE)
