# Supplementary figures and images for: Applying a New REFINE Approach in Zymomonas mobilis Identifies Novel sRNAs That Confer Improved Stress Tolerance Phenotypes
Source: Front Microbiol. 2020 Jan 10;10:2987. doi: 10.3389/fmicb.2019.02987 (PMC6970203; doi:10.3389/fmicb.2019.02987)

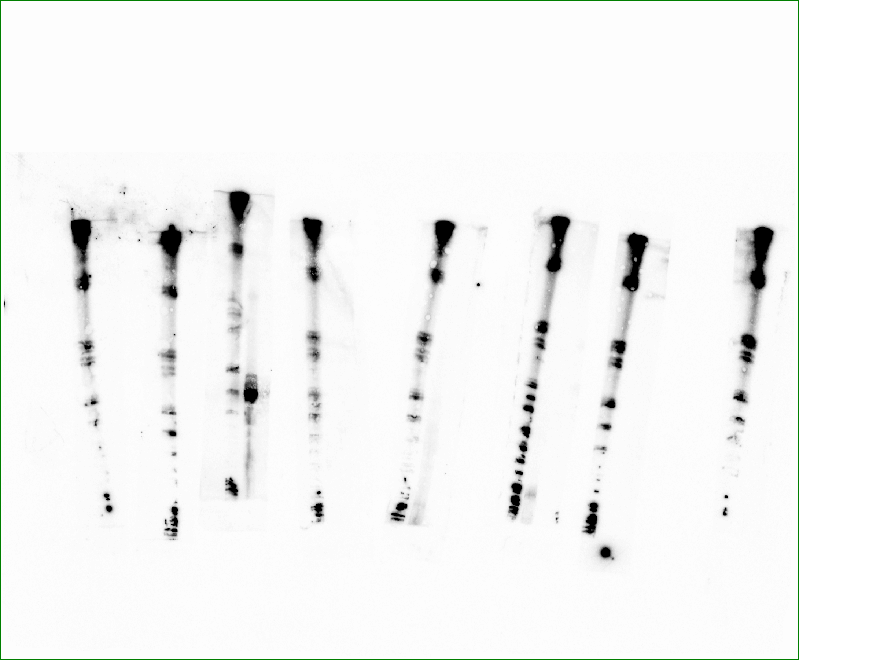

Supplement: DATA SHEET S1 — Full images of northern blots for each confirmed sRNA identified by REFINE. In each blot, the first lane includes 30 μg total RNA of wild-type Z. mobilis 8b grown in anaerobic conditions and collected in stationary phase. The second lane includes ΦX174 DNA/HinfI Dephosphorylated Markers. (A) negsRNA302, (B) negsRNA355, (C) possRNA305, (D) negsRNA78, (E) possRNA223, and (F) negsRNA212. [file Data_Sheet_1.ZIP › sRNA original Northerns/negsRNA212.bmp]

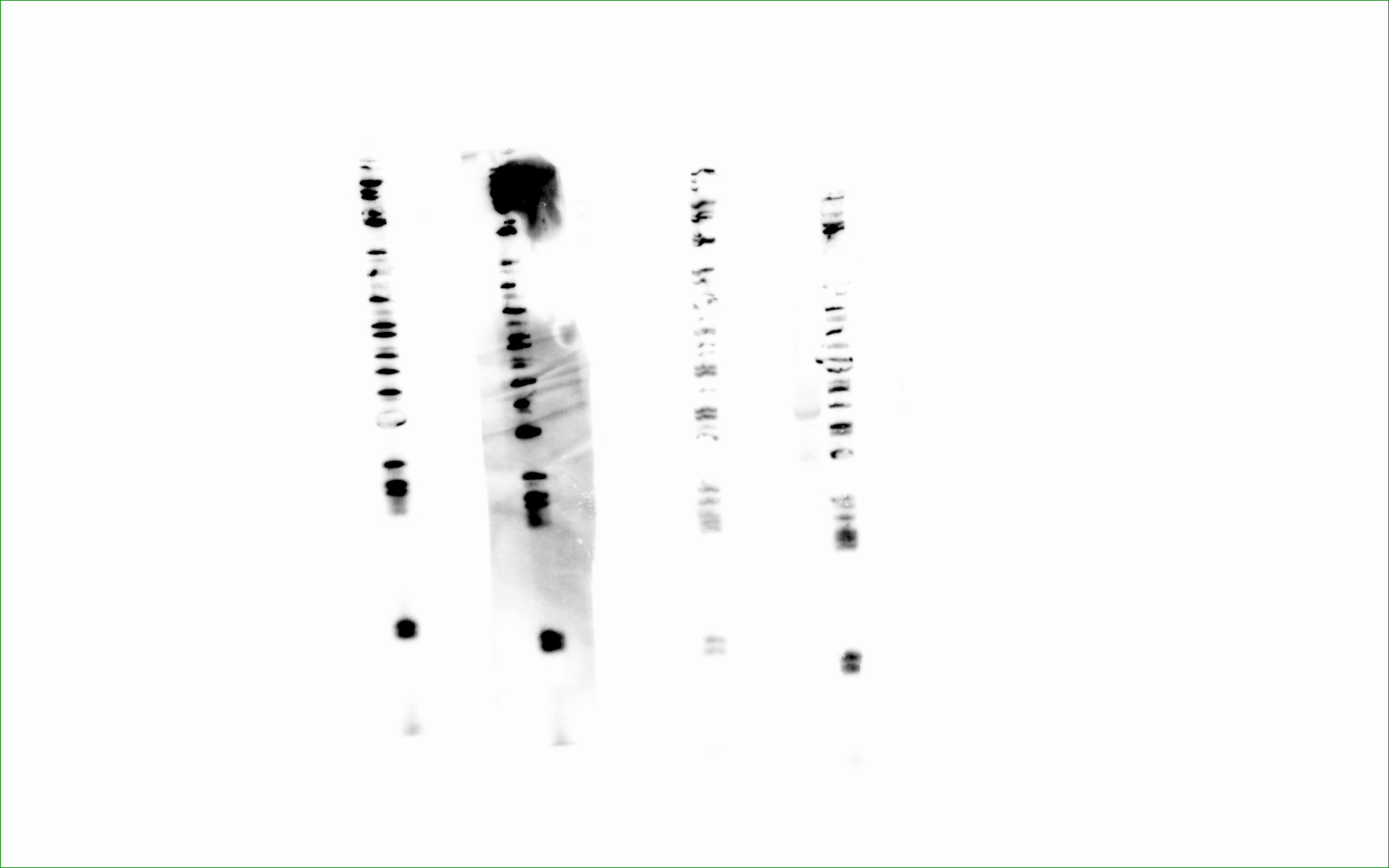

Supplement: DATA SHEET S1 — Full images of northern blots for each confirmed sRNA identified by REFINE. In each blot, the first lane includes 30 μg total RNA of wild-type Z. mobilis 8b grown in anaerobic conditions and collected in stationary phase. The second lane includes ΦX174 DNA/HinfI Dephosphorylated Markers. (A) negsRNA302, (B) negsRNA355, (C) possRNA305, (D) negsRNA78, (E) possRNA223, and (F) negsRNA212. [file Data_Sheet_1.ZIP › sRNA original Northerns/negsRNA302.bmp]

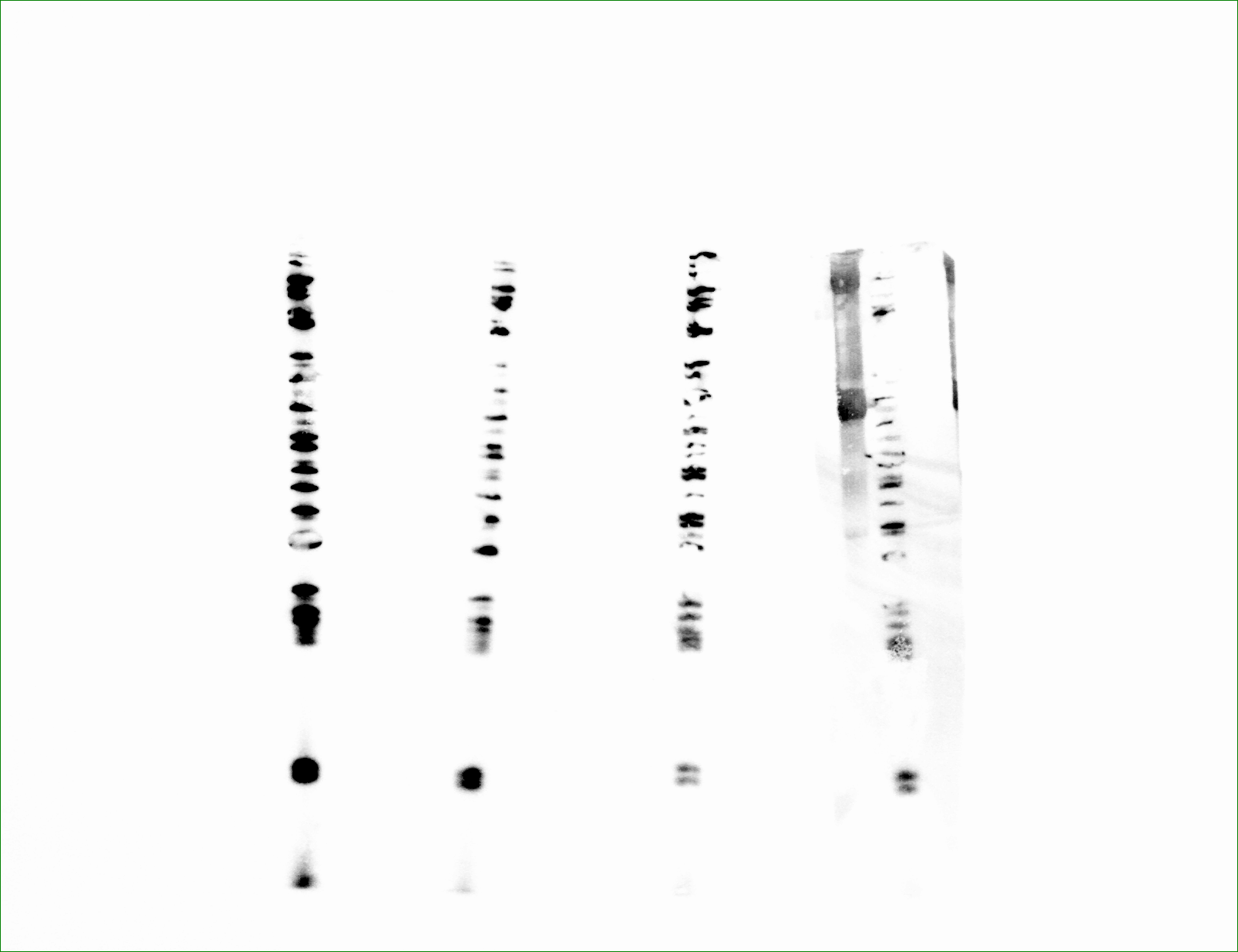

Supplement: DATA SHEET S1 — Full images of northern blots for each confirmed sRNA identified by REFINE. In each blot, the first lane includes 30 μg total RNA of wild-type Z. mobilis 8b grown in anaerobic conditions and collected in stationary phase. The second lane includes ΦX174 DNA/HinfI Dephosphorylated Markers. (A) negsRNA302, (B) negsRNA355, (C) possRNA305, (D) negsRNA78, (E) possRNA223, and (F) negsRNA212. [file Data_Sheet_1.ZIP › sRNA original Northerns/negsRNA355.bmp]

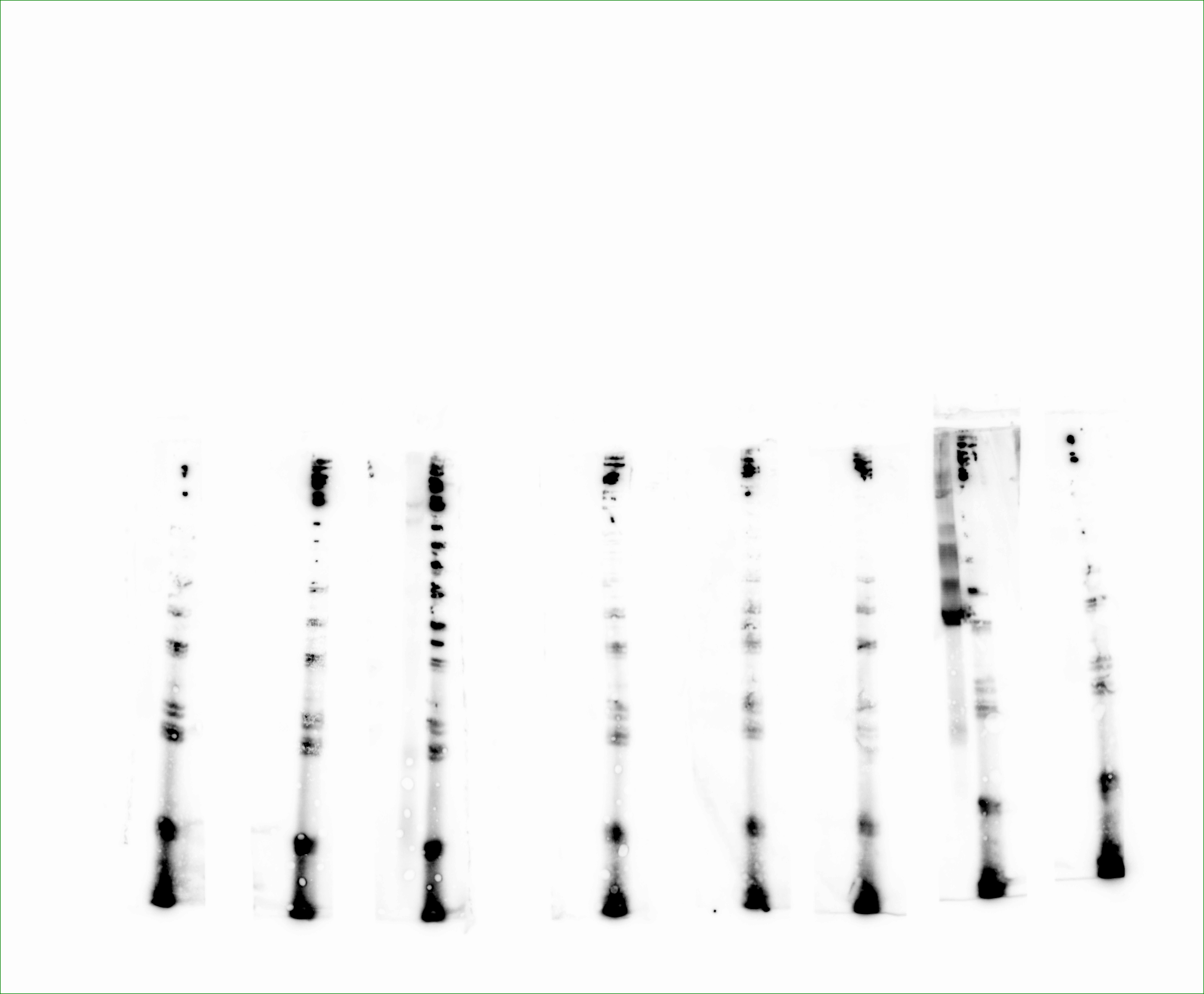

Supplement: DATA SHEET S1 — Full images of northern blots for each confirmed sRNA identified by REFINE. In each blot, the first lane includes 30 μg total RNA of wild-type Z. mobilis 8b grown in anaerobic conditions and collected in stationary phase. The second lane includes ΦX174 DNA/HinfI Dephosphorylated Markers. (A) negsRNA302, (B) negsRNA355, (C) possRNA305, (D) negsRNA78, (E) possRNA223, and (F) negsRNA212. [file Data_Sheet_1.ZIP › sRNA original Northerns/negsRNA78.bmp]
